# Deletion of the Pseudorabies Virus gE/gI-US9p complex disrupts kinesin KIF1A and KIF5C recruitment during egress, and alters the properties of microtubule-dependent transport *in vitro*

**DOI:** 10.1371/journal.ppat.1008597

**Published:** 2020-06-08

**Authors:** Drishya Diwaker, John W. Murray, Jenna Barnes, Allan W. Wolkoff, Duncan W. Wilson

**Affiliations:** 1 Department of Developmental and Molecular Biology, Albert Einstein College of Medicine, Bronx, New York, New York, United States of America; 2 Department of Anatomy and Structural Biology, Albert Einstein College of Medicine, Bronx, New York, New York, United States of America; 3 Marion Bessin Liver Research Center, Albert Einstein College of Medicine, Bronx, New York, New York, United States of America; 4 Dominick P. Purpura Department of Neuroscience, Albert Einstein College of Medicine, Bronx, New York, New York, United States of America; Princeton University, UNITED STATES

## Abstract

During infection of neurons by alphaherpesviruses including Pseudorabies virus (PRV) and Herpes simplex virus type 1 (HSV-1) viral nucleocapsids assemble in the cell nucleus, become enveloped in the cell body then traffic into and down axons to nerve termini for spread to adjacent epithelia. The viral membrane protein US9p and the membrane glycoprotein heterodimer gE/gI play critical roles in anterograde spread of both HSV-1 and PRV, and several models exist to explain their function. Biochemical studies suggest that PRV US9p associates with the kinesin-3 motor KIF1A in a gE/gI-stimulated manner, and the gE/gI-US9p complex has been proposed to recruit KIF1A to PRV for microtubule-mediated anterograde trafficking into or along the axon. However, as loss of gE/gI-US9p essentially abolishes delivery of alphaherpesviruses to the axon it is difficult to determine the microtubule-dependent trafficking properties and motor-composition of Δ(gE/gI−US9p) particles. Alternatively, studies in HSV-1 have suggested that gE/gI and US9p are required for the appearance of virions in the axon because they act upstream, to help assemble enveloped virions in the cell body. We prepared Δ(gE/gI-US9p) mutant, and control parental PRV particles from differentiated cultured neuronal or porcine kidney epithelial cells and quantitated the efficiency of virion assembly, the properties of microtubule-dependent transport and the ability of viral particles to recruit kinesin motors. We find that loss of gE/gI-US9p has no significant effect upon PRV particle assembly but leads to greatly diminished plus end-directed traffic, and enhanced minus end-directed and bidirectional movement along microtubules. PRV particles prepared from infected differentiated mouse CAD neurons were found to be associated with either kinesin KIF1A or kinesin KIF5C, but not both. Loss of gE/gI-US9p resulted in failure to recruit KIF1A and KF5C, but did not affect dynein binding. Unexpectedly, while KIF5C was expressed in undifferentiated and differentiated CAD neurons it was only found associated with PRV particles prepared from differentiated cells.

## Author summary

Alphaherpesviruses including Pseudorabies virus (PRV) and Herpes simplex virus type 1 (HSV-1) are pathogens of the nervous system. They replicate in the nerve cell body then travel great distances along axons to reach nerve termini and spread to adjacent epithelial cells. Virally encoded membrane proteins US9p and the heterodimer gE/gI play critical roles in anterograde spread for both HSV-1 and PRV, however there is disagreement over their roles. Here we compared mutant PRV virions lacking gE/gI-US9p with a parental control strain, and quantitated the efficiency of virion assembly, the properties of microtubule-dependent transport and the ability of viral particles to recruit kinesins. We find that loss of gE/gI-US9p has no effect upon PRV particle assembly but that mutant virions demonstrate greatly reduced transport to the plus-ends of microtubules, enhanced minus-end traffic and greater frequency of reversal in their motion. These changes are accompanied by loss of both kinesin KIF1A and kinesin KIF5C from the mutant, motors that we find associated with distinct populations of the parental virus. We propose a model in which gE/gI-US9p binds KIF1A to ensure efficient plus end-mediated transport and delivery of PRV particles to a location at which KIF5C is subsequently recruited.

## Introduction

During infection of neurons, alphaherpesviruses including Herpes simplex virus type 1 (HSV-1) and Pseudorabies virus (PRV) travel from their site of capsid assembly and DNA packaging in the infected cell nucleus through the cell body, into the axon and eventually traffic to axon termini [1–3]. Anterograde transport of viral particles down the axon, and probably also delivery of virions from the cell body into the axon, is accomplished by kinesin-mediated trafficking along microtubules, and KIF1A and KIF5 (members of the kinesin-3 and kinesin-1 motor families respectively) [4–6] appear to play important roles [1, 3]. KIF1A is brain specific, one of the fastest known anterograde motors [4, 5] and normally transports synaptic vesicle precursors containing synaptophysin, synaptotagmin and Rab3A [4, 6]. Although KIF1A exists as a monomer [4] it is highly processive when multiple copies of the motor cooperate in teams [7]. KIF5 is expressed as three closely related subtypes, the ubiquitously expressed KIF5B and neuron-specific KIF5A and KIF5C [4]. The KIF5 motor is a homo or heterodimer of two KIF5 chains (termed the kinesin heavy chains [KHC]) and often additional subunits including the kinesin light chains (KLC) [5] or milton, which are thought to serve as cargo adaptors [5, 8]. Since they consist of KHC dimers, KIF5 motors have the capacity to move high-load cargo [7] including mitochondria, lysosomes, and synaptic vesicle precursors along axons [4–6] and ~1000S mRNA-containing ribonucleoprotein granules within dendrites [4, 9].

Three virally encoded membrane proteins are known to be important in the anterograde spread of infection for both HSV-1 and PRV [2, 3, 10–14]. US9p is a small, non-glycosylated lipid raft-associated type II membrane protein while glycoproteins E and I (gE and gI) are type I membrane proteins that appear to always function in the context of a gE/gI heterodimer [3, 10, 15–18]. For HSV-1 gE/gI and US9p are important for anterograde spread within the nervous system [19–22] in an apparently cooperative and partially redundant fashion; loss of US9p reduces the numbers of HSV-1 particles in distal axons by about 50% [15, 23] but simultaneous loss of US9p and gE/gI abolishes delivery to axons almost completely [24]. In contrast, PRV strains with the US9 gene deleted are completely defective for axonal sorting of viral particles and exhibit no anterograde spread of infection [16–18, 25].

There is strong evidence that at least one role for gE/gI and US9p in HSV-1 and PRV anterograde spread is to provide a kinesin-receptor for the anterograde transport of organelles carrying enveloped virion cargo. In infected primary rat superior cervical ganglia (SCG) neurons gE/gI and US9p localize to the *trans* Golgi network (TGN) in the cell body and to PRV and HSV-1 capsid-containing virions undergoing anterograde transport in the axon [26]. The PRV and HSV-1 gE/gI and US9p proteins exhibit a similar localization pattern even in the absence of viral infection, showing that the trafficking properties of gE/gI-US9p alone resembles that of egressing virions [26]. Biochemical studies indicate that PRV gE/gI and US9p form a tripartite complex within detergent resistant membrane (DRM) lipid rafts in infected SCG neurons, and serves to also recruit KIF1A into these DRMs [26]. US9p is likely to be the subunit of the gE/gI-US9p complex that directly binds motors since PRV US9p copurifies with KIF1A in extracts prepared from PRV infected PC12 cells [27] though gE/gI stimulates or stabilizes the US9p/KIF1A interaction [28]. A modulatory effect of gE/gI upon US9p-mediated KIF1A recruitment or activation is also suggested by the finding that PRV gI accelerates the KIF1A-mediated motility of PRV particles along axons or, alternatively, inhibits opposed dynein-mediated retrograde transport [26].

Together these data lead to a model in which a tripartite gE/gI-US9p complex recruits KIF1A for alphaherpesvirus transport into and perhaps also along axons, and is consistent with the observation that anterograde-trafficking PRV and HSV-1 capsids co-translocate with KIF1A, but not with KIF5B, in the axons of infected rat SCG neurons [26, 27]. That the primary purpose of gE/gI-US9p is to recruit (and perhaps regulate) KIF1A is suggested by the finding that the axonal sorting, transport, and anterograde spread defect of Δ(gE/gI-US9p) PRV virions can be largely suppressed by directing the recruitment of KIF1A to PRV particles by artificial means [26].

Several lines of evidence are nevertheless inconsistent with a role for the gE/gI-US9p-KIF1A complex in alphaherpesvirus transport along the axon. While deletion of US9 clearly results in fewer PRV particles entering the axon, those rare virions that do so exhibit microtubule-based anterograde transport kinetics that are indistinguishable from wild type [29]. Similar data were reported for HSV-1 where simultaneous loss of HSV-1 US9p and gE/gI led to severely diminished numbers of trafficking particles within the axon, but rare axonal virions exhibit normal rates of anterograde traffic [15, 24]. Moreover, anterograde-trafficking HSV-1 particles in the neurites of cultured mouse CAD neurons colocalize with KIF5C and with KIF5C-borne cargo, but not with KIF1A [30]. Similarly, HSV-1 trafficking in these CAD neurites was inhibited by silencing of KIF5A, -5B, and -5C, or the kinesin light chains KLC1 and KLC2, but not by silencing of KIF1A [30].

One explanation for these findings is that the reduction in anterograde spread seen for ΔUS9p PRV and Δ(gE/gI-US9p) HSV-1 strains is due to a defect in KIF1A-mediated sorting of viral particles within the soma, or from the soma into axons, rather than a defect in microtubule-based anterograde transport within the axon itself [3, 29]. In this model gE/gI-US9p-KIF1A would ensure delivery of viral cargo to the axon and then KIF1A would be exchanged for, or supplemented by, KIF5 family members [4, 5, 26]. The mechanism by which kinesin-1 motors might be recruited to alphaherpesvirus particles to drive axonal transport remains unknown although amyloid precursor protein (APP), a candidate kinesin-1/KIF5 receptor [4, 31, 32] has been found associated with anterograde-trafficking HSV-1 in a squid giant axon transport model [33], in epithelial cells [34] and with KIF5C-bound HSV-1 particles in CAD neurites [30]. Moreover, the US9p of HSV-1 has been reported to directly interact with the C-terminal tail of KIF5B [35], consistent with the possibility that it recruits kinesin-1 motors for transport along axons. Nevertheless, deletion of US9 does not appear to affect the rate of trafficking of axonal HSV-1 particles *in vivo* [24, 30].

Other models have been proposed to account for the failure of Δ(gE/gI-US9p) PRV and HSV-1 particles to travel from the cell body into the neurites or axons of neuronal cells. For HSV-1 it has been shown that gE/gI and US9p cooperate to drive envelopment of cytoplasmic capsids in the cell body, generating the enveloped viral cargo which is subsequently delivered to the axon [15, 36]. US9p has also been implicated in capsid envelopment at the growth cones in distal axons [37]. Alternatively, or in addition, the cytoplasmic tails of gE/gI or US9p projecting from the surface of organelles such as the TGN [26, 38] have been suggested to “load” kinesins onto viral particles for their subsequent transport [15, 24]. However the details of such a mechanism remain to be established.

Since loss of gE/gI-US9p essentially abolishes delivery of alphaherpesvirus particles to the axon it is extremely difficult to determine the microtubule-dependent trafficking properties and motor-composition of ΔgE/gI-ΔUS9p virions [15, 24, 26, 29]. For this reason, while PRV US9p clearly associates with KIF1A in biochemical extracts [26–28] it remains to be shown whether loss of the gE/gI-US9p complex affects the recruitment of kinesins to trafficking PRV particles. Similarly, it is unclear whether loss of gE/gI and US9p affects the assembly of enveloped PRV particles prior to their sorting into the axon [36] as has been proposed for HSV-1 [15, 36, 37]. In this study we prepared Δ(gE/gI-US9p) PRV particles, and those of a parental control strain, from the cytoplasm of differentiated cultured neuronal cells or porcine kidney epithelial cells. We then quantitated the efficiency of virion assembly, analyzed the transport properties of mutant virions using an *in vitro* microtubule-dependent trafficking assay and tested the ability of mutant viral particles to recruit the motors KIF1A, KIF5C and dynein.

## Results

### Loss of gE/gI-US9p has no effect on the numbers of membrane-associated and infectious PRV particles assembled in porcine kidney epithelia PK15 or mouse neuronal CAD cells

PRV GS4284 is a Becker-derived Pseudorabies virus strain that expresses a UL25mCherry capsid fusion protein but is otherwise genetically wild type and is referred to as the “P” (Parental) strain throughout this study. PRV GS6090 is identical to GS4284 except for deletion of genes US7 (encoding gI), US8 (encoding gE) and US9, and is here referred to as the “M” (mutant) strain. CAD cells are a derivative of a mouse catecholaminergic central nervous system cell line that upon differentiation express multiple neuronal markers and develop long neurites analogous to axons [39–41]. Upon HSV-1 infection and replication in the cell body these neurites deny access to anterograde-trafficking HSV-1 particles if they lack gE/gI or US9p [15]. These cells therefore provide an attractive system to model the process of gE/gI-US9p-dependent trafficking. In many of the studies described below we compare the assembly and trafficking of CAD cell-derived PRV particles with virions isolated from porcine kidney epithelial PK15 cells.

**Fig 1:**
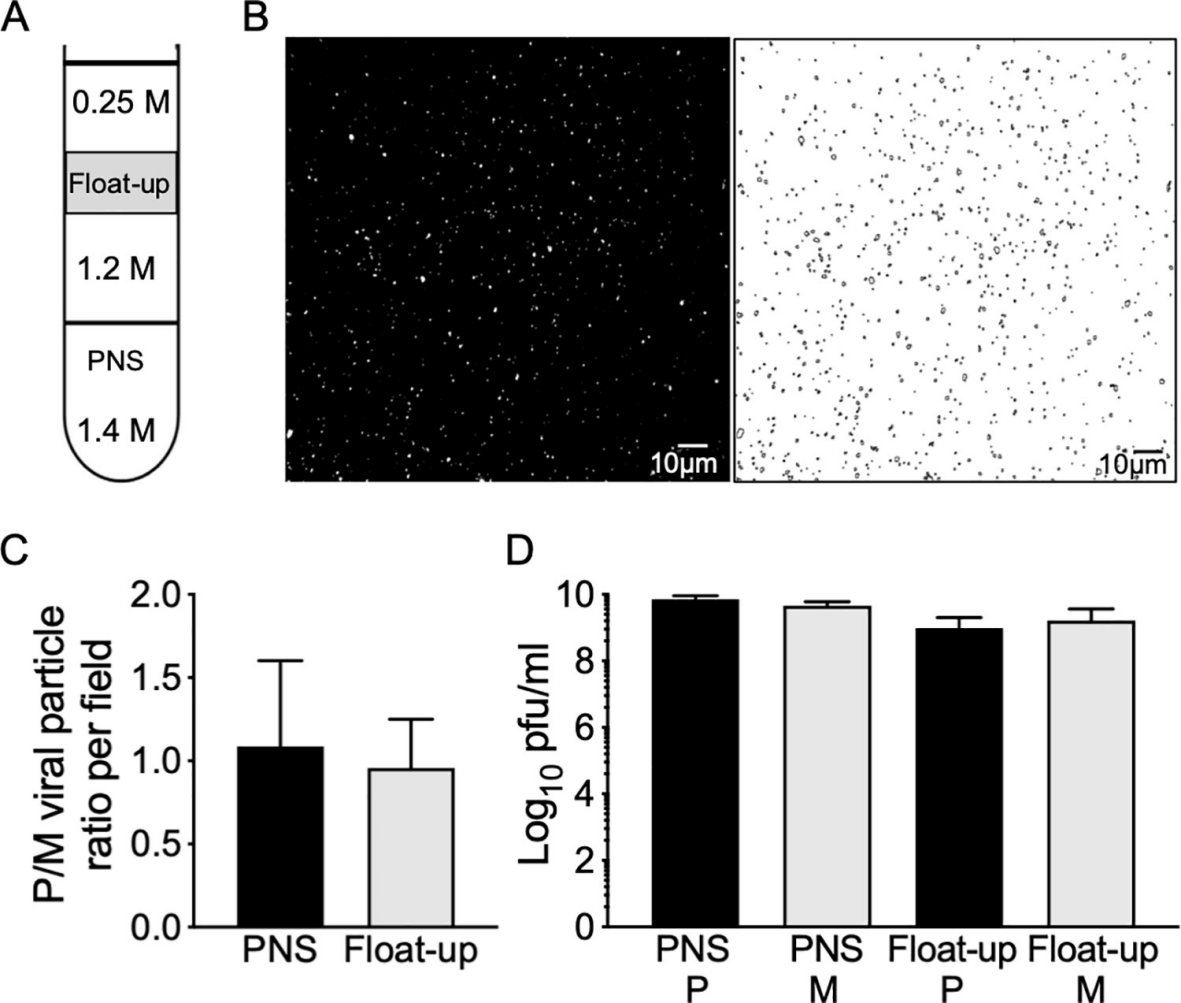
Quantitation of particles and infectious virions produced by Δ(gE/gI-US9p) and parental PRV in PK15 cells. PK15 cells were infected by parental (P) and Δ(gE/gI-US9p) mutant (M) PRV. A PNS was then prepared, adjusted to a final sucrose concentration of 1.4M, overlaid with 1.2M and 0.25M sucrose layers then subjected to density gradient flotation to isolate buoyant cytoplasmic particles. **(A)** Gradient conditions showing molarity of sucrose in each cushion and location of the buoyant float-up fraction (grey rectangle). **(B)** Left half of panel shows microscopic field of a sample of the float-up fraction viewed in the red channel to image particles labeled with the mCherry-tagged UL25p capsid subunit. Right half of panel shows Fiji-processed image used for quantitation. **(C)** Relative numbers of mCherry-fluorescent P and M particles in microscopic fields of PNS (black bar) or float-up (grey bar) samples. Y axis corresponds to the ratio of numbers of P and M PRV particles counted in microscopic fields imaged and analyzed as in panel B. Plotted ratios are the mean and standard deviation from the mean for three independently prepared sets of P and M extracts, each of which was quantitated using at least seven independently prepared microscopic fields. Total particle numbers counted were 285,226 (P) and 185,576 (M) (PNS) and 10,069 (P) and 11,652 (M) (float-up). **(D**) Samples of PNS and float-up from P (black bars) and M (grey bars) infected cells were sonicated and titrated onto preformed Vero cell monolayers. Plotted values are the mean and standard deviation from the mean for titers of PNS and float-up samples prepared from three independently derived sets of P and M cell extracts, each titered in duplicate.

**Fig 2:**
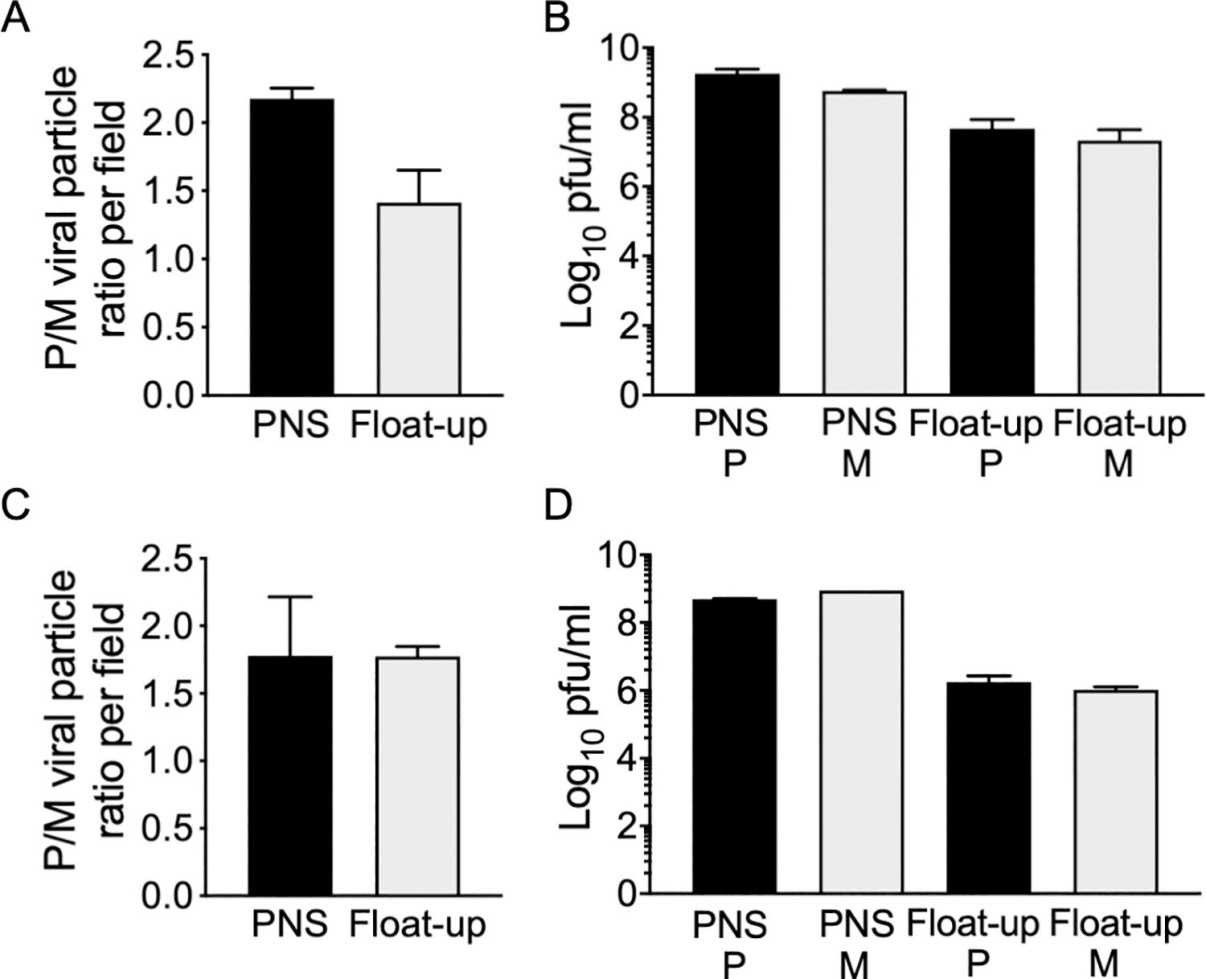
Quantitation of particles and infectious virions produced by Δ(gE/gI-US9p) and parental PRV in CAD cells. **(A, C)** Relative virus count/field plotted as ratio of PRV P to M particles in PNS or float-up fractions from undifferentiated CAD cells and differentiated CAD cells, respectively. Quantitation was as in Fig 1C. Plotted ratios are the mean and standard deviation from the mean for three independently prepared sets of P and M extracts, each of which was quantitated using at least seven independently prepared microscopic fields. Total particle numbers counted were 19,541 (P) and 8,971 (M) (PNS) and 37,867 (P) and 27,769 (M) (float-up) for undifferentiated CAD cells, 20,608 (P) and 12,846 (M) (PNS) and 16,224 (P) and 8,330 (M) (float-up) for differentiated CAD cells. **(B, D)** Plaque assay of P and M PNS and float-up from undifferentiated CAD cells and differentiated CAD cells, respectively. Titers were determined exactly as in Fig 1D. Plotted values are the mean and standard deviation from the mean for titers of PNS and float-up samples prepared from three independently derived sets of P and M cell extracts, each titered in duplicate.

The gE/gI heterodimer plays important roles in HSV-1 capsid cytoplasmic envelopment in epithelial [42, 43] and neuronal cells, in the latter case in cooperation with US9p [15, 36, 37]. Inefficient capsid envelopment, and ensuing reduction in numbers of enveloped virions in the neuronal cell body, provides one explanation for the failure of ΔgE/gI and ΔUS9p HSV-1 particles to be efficiently delivered to axons and neurites during egress [15, 36]. To test this possibility for PRV we quantitated enveloped virion assembly in the presence and absence of gE/gI and US9p following viral replication in epithelial cells and neurons. We infected porcine kidney PK15 cells with P and M PRV strains then prepared post nuclear supernatants (PNS) and membrane-associated buoyant “float-up” fractions (Fig 1A). Viral particles were imaged by capsid-associated mCherry fluorescence in microscopic fields (Fig 1B) and the numbers of cytoplasmic and membrane-associated particles were counted (Fig 1C). Cytoplasmic and membrane-associated fractions were also titered to determine numbers of infectious particles. We found that loss of gE/gI and US9p had no significant effect on the numbers of membrane-associated PRV particles assembled, and no effect upon PFU production in these epithelial cells (Fig 1D). We next performed similar experiments using undifferentiated and differentiated CAD cells [39], a cell line in which a ΔgE/ΔUS9p HSV-1 strain exhibits defective cytoplasmic envelopment [15]. We found that loss of gE/gI and US9p resulted in a less than 2-fold reduction in numbers of membrane-associated PRV particles assembled in undifferentiated or differentiated CAD cells (Fig 2A and 2C), and had no significant effect upon yields of infectious virions (Fig 2B and 2D). We conclude that, for PRV under these replication conditions, loss of gE/gI and US9p do not diminish the numbers of membrane-associated and infectious virions that are assembled in the cell body of PK15 epithelial cells or CAD neurons.

### Loss of gE/gI-US9p changes the *in vitro* trafficking properties of membrane-associated PRV particles

**Fig 3:**
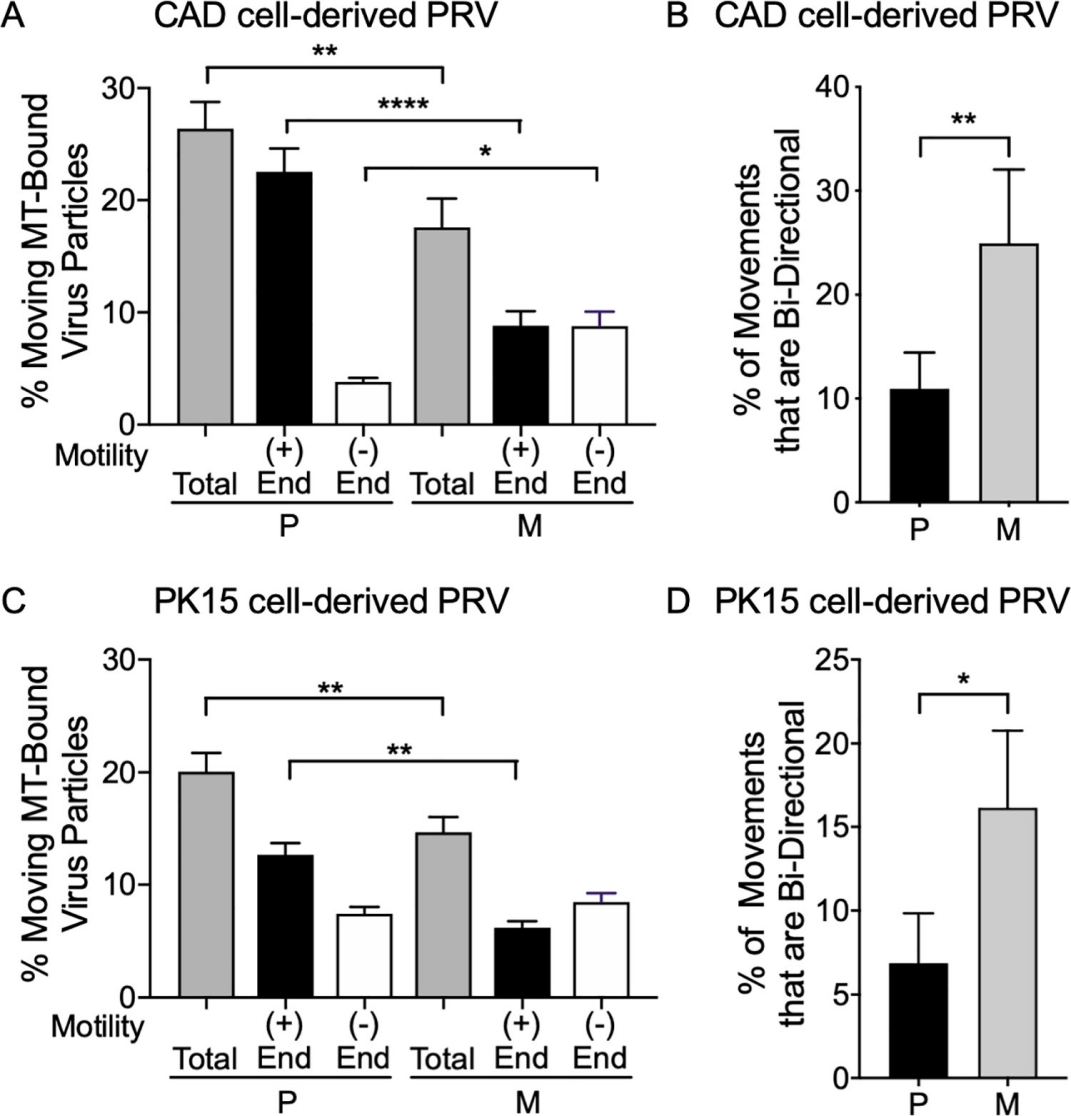
Loss of gE/gI-US9p reduces microtubule-mediated trafficking and increases frequency of minus end and bidirectional motion. Float-up organellar fractions from P and M PRV-infected differentiated CAD or PK15 cells were bound to microtubules in a microscopic imaging chamber and their frequency and direction of *in vitro* motility determined. **(A)** Motile P and M PRV particles prepared from infected differentiated CAD cells. Total motile particles (grey bars) are expressed as a % of the number of viral particles bound to microtubules. Plotted values are the mean and standard deviation from the mean for 89 P and 82 M motile particles. In a separate experiment the motility of P and M PRV particles towards the plus end (+, black bars) or minus end (-, white bars) of directionally labeled microtubules was determined. Plotted values are the mean and standard deviation from the mean for 81 P and 54 M motile particles. Particles exhibiting bidirectional motion were excluded from this count. **(B)** P and M PRV particles derived from differentiated CAD cell float-up fractions that exhibited bidirectional motion on microtubules. Plotted values are mean and standard deviation from the mean for 9 P and 15 M motile particles. **(C, D)** Similar to (A, B) but testing motility of PRV particles in float-up fractions prepared from PK15 cells. Plotted values are mean and standard deviation from the mean for 99 P and 84 M motile particles (C) and 5 P and 13 M motile particles (D). * P≤ 0.05, ** P≤ 0.01, **** P≤ 0.0001.

US9p and gE/gI are believed to cooperate to provide a KIF1A receptor activity for transport of PRV particles along the axon [26–28]. However, the kinetics of microtubule-based anterograde transport of the rare ΔUS9 PRV particles that reach the axon are indistinguishable from wild type virions, suggesting it is more likely that gE/gI and US9p instead cooperate to sort PRV particles into the axon [29]. To test whether the failure of Δ(gE/gI-US9p) PRV particle delivery to the axon could itself be due to changes in microtubule-mediated transport we used our previously established assay that reconstitutes microtubule-dependent trafficking of HSV-1 and PRV particle *in vitro* [44–46]. We prepared P and M membrane-associated PRV particles from infected differentiated CAD and PK15 cells and examined their ability to traffic along fluorescently labeled microtubules following addition of ATP. Loss of gE/gI and US9p reduced the frequency with which CAD-derived PRV particles were capable of trafficking along microtubules *in vitro* by one third (Fig 3A grey bars). Similarly, M PRV particles generated by replication in PK15 cells showed a reduction in the frequency of trafficking particles by about one quarter compared to P PRV (Fig 3C grey bars).

Our *in vitro* system simultaneously reconstitutes plus end-directed and minus end-directed transport of alphaherpesviruses along microtubules [44, 46]. Loss of gE/gI and US9p could therefore be reducing overall viral transport by affecting viral traffic in either, or both, directions. We therefore prepared directionally-labeled fluorescent microtubules, whose minus ends can be identified based on their more dimly fluorescent labeling intensity (S1 Fig). The direction of motion of fluorescent viral particles towards the plus or minus ends of the microtubule can then be readily determined (S1 Fig) and scored (S2 Fig). P PRV particles isolated from differentiated CAD cells were found to move predominantly to the plus ends of microtubules (~85% of total motile virions were plus end-directed, Fig 3A black and white bars) as expected for egressing virions. Loss of gE/gI and US9p reduced the numbers of PRV particles travelling in the plus direction by almost two thirds but more than doubled the numbers of particles moving to the minus end (Fig 3A black and white bars). Similar changes were seen for virions isolated from PK15 epithelial cells; about two thirds of P PRV particles were plus end-directed but this number fell by half upon deletion of gE/gI-US9p. However, in this case changes in minus end-directed motion were not statistically significant (Fig 3C black and white bars).

Alphaherpesviruses are known to exhibit bidirectional transport during overall anterograde spread [3, 47] suggesting that both plus and minus end-directed motors are present simultaneously on the viral particles. We reasoned that if deletion of gE/gI-US9p results in fewer (or fewer *active*) plus end-directed motors on the viral particles, then individual virions might exhibit an increased likelihood of bidirectional motion along microtubules. We therefore scored PRV particles that changed their direction of motion along microtubules during the course of our assay. For P PRV particles bidirectional motion was rare; 10% and 7% of motile P particles following isolation from differentiated CAD and PK15 cells respectively (Fig 3B and 3D black bars). However, the numbers of bidirectional particles increased two to three fold upon loss of gE/gI-US9p (Fig 3B and 3D grey bars). Together our data suggest that loss of gE/gI-US9p perturbs the normal balance of plus and minus end-directed motion, reducing the numbers of PRV particles that exhibit sustained anterograde transport.

### Effect of gE/gI and US9p deletion on Kinesin-3 (KIF1A) recruitment

**Fig 4:**
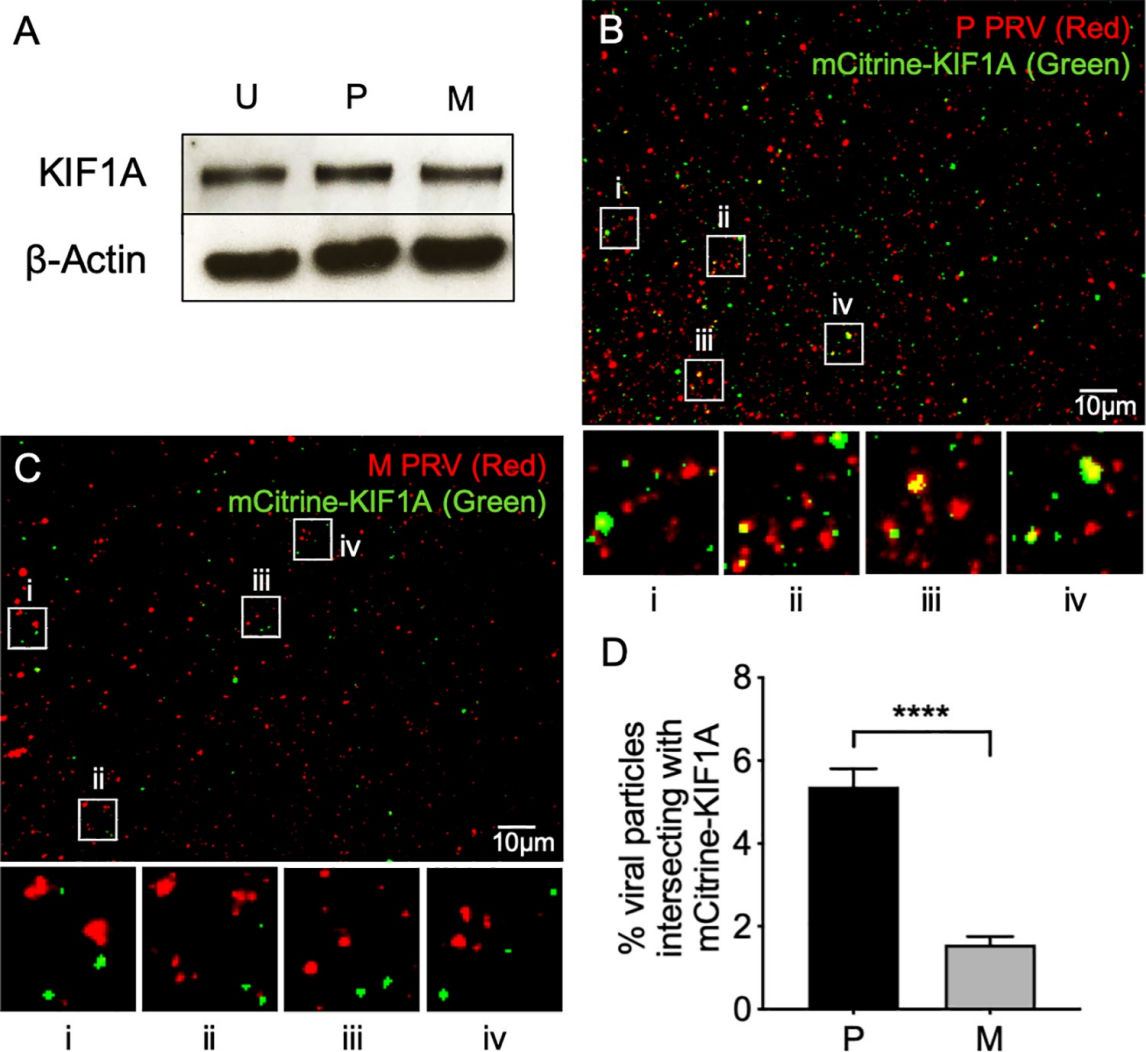
Loss of gE/gI and US9p results in failure of PRV particles to colocalize with KIF1A. **(A)** CAD cells were differentiated and infected with P or M PRV strains or uninfected (U). Float-up fractions were prepared as in Fig 1 then Western blotted for KIF1A or β−actin as a loading control. **(B)** CAD cells were transfected to express mCitrine-KIF1A then differentiated and infected with P PRV, float-up fractions prepared and viewed in the red (mCherry-tagged capsids) and green (mCitrine-KIF1A) channels and a merged image shown. Four boxed regions (i-iv) were magnified 5-fold and are shown at the bottom of the panel. **(C)** Identical to (B) except infection was with the M strain of PRV. In all cases control extracts were prepared under identical conditions from PRV-infected cells that were not expressing mCitrine-KIF1A, in order to establish imaging conditions rendering any mCitrine-KIF1A-independent fluorescence undetectable. The individual channel images used to construct the merged panels B and C are shown in inverted black and white in S3 Fig. **(D)** Numbers of P and M red fluorescent particles that exhibit mCitrine-KIF1A fluorescence as a percent of total particles in the field, derived from images similar to those in (B) and (C). Plotted values are mean and standard deviation from the mean for 22,784 P and 14,908 M particles, respectively.

Loss of plus end-directed motion, and increased frequency of reversals in direction (Fig 3) suggest that M PRV particles are compromised in their ability to recruit or activate kinesin motors. Because PRV US9p biochemically interacts with KIF1A in a gE/gI-stimulated manner [27, 28] we tested whether loss of gE/gI-US9p affected KIF1A recruitment to trafficking PRV virions. Western blotting demonstrated that PRV-containing buoyant fractions contained similar levels of endogenous KIF1A protein whether prepared from control uninfected, P- or M-infected differentiated CAD cells, suggesting no gross overall changes in KIF1A association with cellular organelles in the absence of gE/gI and US9p during infection (Fig 4A). To test for KIF1A recruitment to P and M PRV particles we attempted to detect endogenous KIF1A by immunocytochemistry, but were unsuccessful. Instead we imaged a fully functional and well characterized fusion between KIF1A and mCitrine fluorescent protein [7, 48] which we delivered to these infected differentiated CAD cells by transfection (see Materials and Methods). While mCitrine-KIF1A was found to decorate a subpopulation of capsid-associated P PRV particles (Fig 4B) this colocalization was significantly diminished in the ΔgE/gI-ΔUS9p mutant virus (Fig 4C). These data are quantitated in Fig 4D.

### Effect of gE/gI and US9p deletion on Kinesin-1 (KIF5C) recruitment

**Fig 5:**
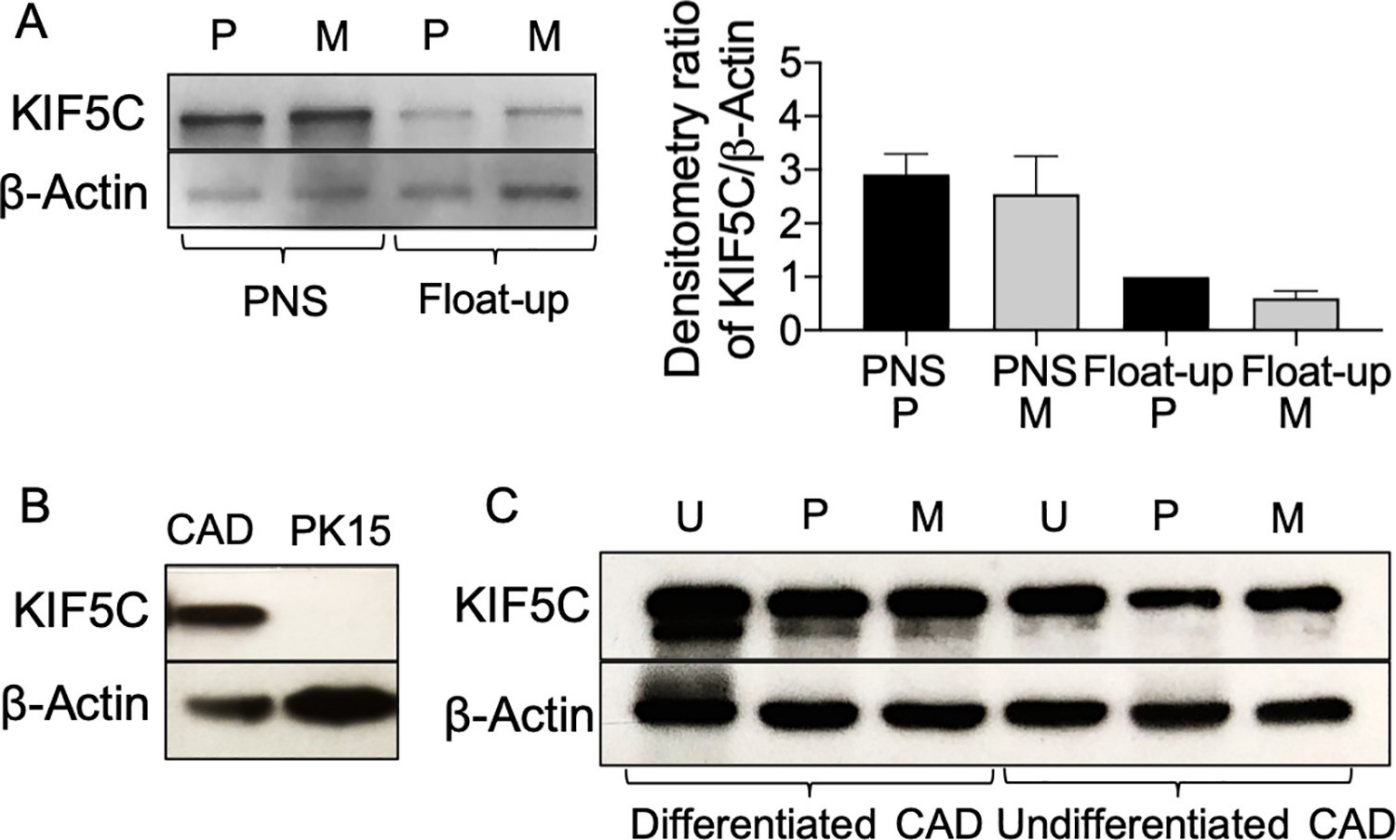
Western blots to demonstrate expression of KIF5C in infected and uninfected cells. **(A)** Left of panel: Western blot of PNS and float-up fractions prepared from P or M PRV-infected differentiated CAD cells. Right of panel: Densitometric analysis of anti-KIF5C and anti-β−actin reactive bands from similar Western blots (data is the mean and range derived from two independently generated blots). **(B**) Western blot of PNS prepared from P PRV-infected PK15 or differentiated CAD cells. **(C)** Western blot of whole cell lysates prepared from differentiated or undifferentiated CAD cells that had been infected with P or M PRV strains or uninfected (U). In all panels the Western blot for β−actin provides a loading control.

KIF5C colocalizes with HSV-1 particles trafficking in the neurites of differentiated CAD cells [30], and US9p of HSV-1 has been reported to directly interact with the C-terminal tail of KIF5B [35]. We therefore next tested whether loss of gE/gI and US9p perturbed recruitment of the neuronal-specific kinesin KIF5C to PRV particles. First we examined overall cellular levels of the KIF5C motor by Western blot (Fig 5). Following infection by P or M strains of PRV, similar levels of KIF5C protein were found in differentiated CAD cell total cytoplasmic extracts (PNS), float-up fractions (Fig 5A) and whole cell extracts (Fig 5C). Levels of KIF5C were slightly diminished in P and M PRV-infected differentiated CADs compared to an equivalent number of uninfected control cells (Fig 5C). As expected, we did not detect the neuronal-specific KIF5C in a PNS prepared from epithelial PK15 cells (Fig 5B). Interestingly, KIF5C was readily detectable in uninfected or PRV-infected CAD cells whether or not they had been differentiated (Fig 5C).

**Fig 6:**
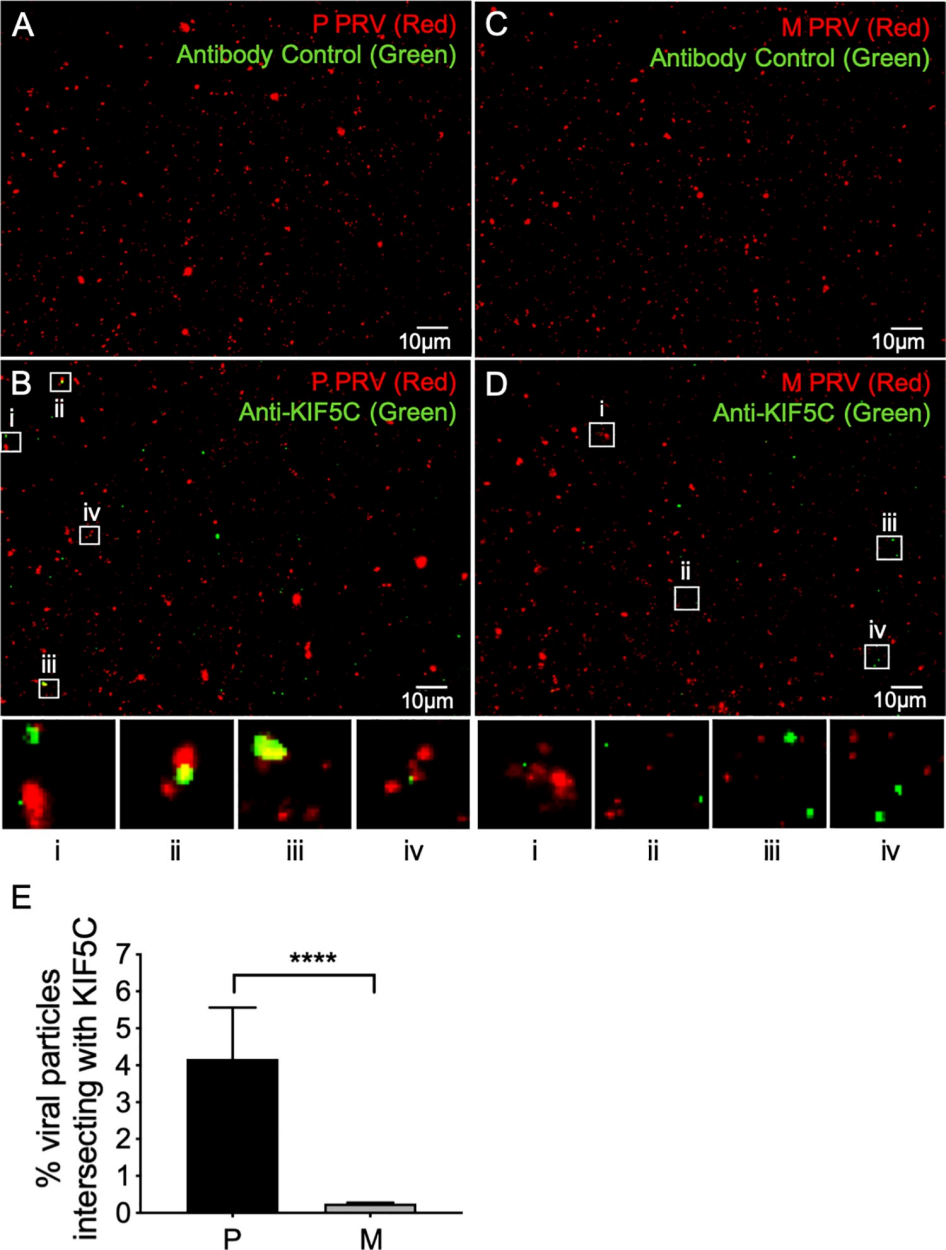
Recruitment of KIF5C to PRV particles requires gE/gI-US9p. Float-up fractions from P or M PRV-infected differentiated CAD cells were immunostained with a rabbit anti-KIF5C or control IgG (as indicated at top of each panel), imaged in the red (mCherry-tagged capsids) and green (anti-rabbit antibody immunofluorescence) channels and images merged. **(A, B)** Fields of float-up particles from a P PRV infection stained with control IgG and anti-KIF5C IgG respectively. **(C, D)** Fields of float-up particles from a M PRV infection stained with control IgG and anti-KIF5C IgG respectively. Four boxed regions (i-iv) in panels B and D were magnified 5-fold and are shown at the bottom of each panel. The individual channel images used to construct the merged panels in A-D are shown in inverted black and white in S4 Fig and S5 Fig. **(E)** Numbers of P and M red fluorescent particles that exhibit anti-KIF5C staining as a percent of total particles in the field, derived from images similar to those in (B) and (D). Plotted values are mean and standard deviation from the mean for 20,529 P and 16,830 M particles, respectively.

We next examined whether endogenous KIF5C colocalized with PRV capsid-associated particles (Fig 6). Float-up fractions of P PRV were immunostained with a control rabbit IgG (Fig 6A) or anti-KIF5C (Fig 6B). Identical immunostaining was performed for M PRV particles (Fig 6C and 6D). As quantitated in Fig 6E a subpopulation of P PRV particles exhibited bound KIF5C, but KIF5C was essentially undetectable on M PRV particles despite being present in this float up fraction as demonstrated by Western blotting (Fig 5A).

### KIF1A and KIF5C bind to distinct populations of egressing PRV particles

**Fig 7:**
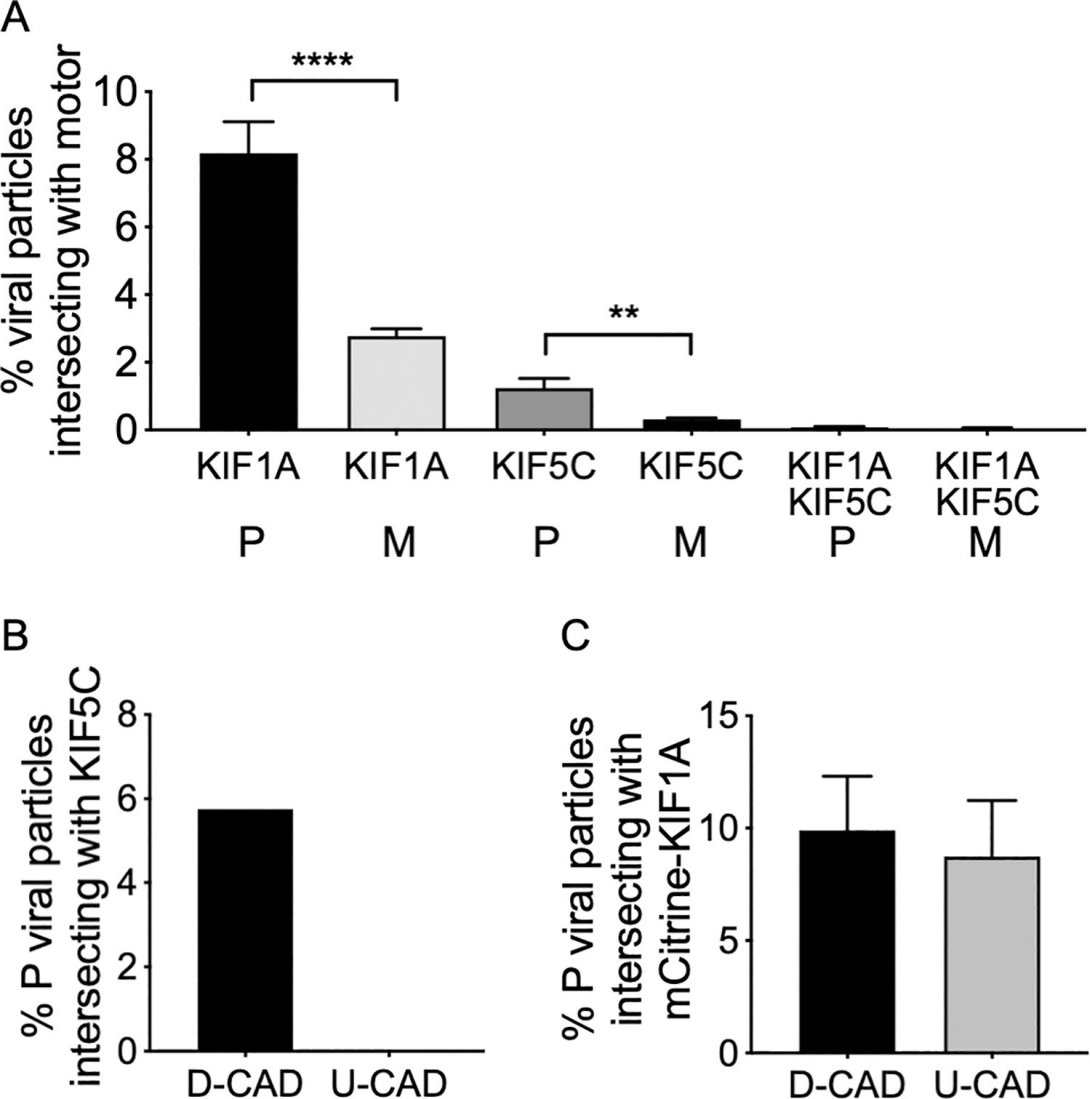
KIF1A and KIF5C label distinct populations of PRV particles, and PRV association with KIF5C requires CAD cell differentiation. **(A)** CAD cells were transfected to express mCitrine-KIF1A, differentiated and infected with P PRV. Float-up fractions were immunostained for KIF5C as in Fig 6 and fields simultaneously imaged for mCherry-tagged capsids (red channel), anti-KIF5C (blue channel) and mCitrine-KIF1A (green channel). Red particles were scored for colocalization with KIF1A, KIF5C or both motors as indicated on the X axis, and colocalization plotted as a percent of total red particles in the field. Plotted values are mean and standard deviation from the mean for 4,007 P and 1,830 M particles each counted from at least 10 microscopic fields. **(B)** Differentiated (D-CAD) or undifferentiated (U-CAD) CAD cells were infected with P PRV, float-up fractions immunostained for KIF5C and numbers of red fluorescent particles that exhibit anti-KIF5C staining determined as in Fig 6. Plotted values are derived from 8,643 and 9,712 red fluorescent particles (prepared from D-CAD and U-CAD cells respectively). **(C)** CAD cells were transfected to express mCitrine-KIF1A and either infected with P PRV or first differentiated then infected. Float-up fractions were prepared and numbers of red fluorescent particles that exhibit mCitrine-KIF1A fluorescence determined as in Fig 4. Plotted values are mean and standard deviation from the mean for 22,405 and 16,794 particles (prepared from D-CAD and U-CAD cells respectively) each counted from 13 microscopic fields.

P PRV particles prepared from differentiated CAD cells were decorated by kinesins KIF1A (Fig 4) and KIF5C (Fig 6A–6E). We next asked whether these motors are present simultaneously on cytoplasmic PRV particles or are instead localized to distinct viral subpopulations. To examine this question we performed anti-KIF5C immunostaining of float-up fractions prepared from PRV-infected differentiated CAD cells transfected to express mCitrine-KIF1A. As seen previously (Fig 4 and Fig 6) KIF1A and KIF5C were each readily detectable on P PRV particles, and loss of gE/gI and US9p reduced the efficiency of recruitment of both motors (Fig 7A). However, for both P and M PRV fewer than one tenth of one percent of capsid-associated particles simultaneously exhibited mCitrine-KIF1A fluorescence and anti-KIF5C immunostaining (Fig 7A). We conclude that KIF1A and KIF5C rarely exist on the same population of PRV particles during egress in differentiated CAD cells, although it remains possible that some PRV particles simultaneously bind both motors at levels too low for us to detect.

### KIF1A, but not KIF5C, binds to PRV particles in undifferentiated CAD cells

We considered the possibility that KIF1A and KIF5C-bound PRV particles might correspond to distinct phases of microtubule-dependent virion transport within the highly polarized environment of the differentiated CAD neuron. If so, the profile of motor recruitment might be altered in undifferentiated CAD cells. To test this, differentiated or undifferentiated CAD cells were infected with P PRV and float-up fractions immunostained for KIF5C. KIF5C labeling of PRV particles was readily detectable in extracts prepared from differentiated CAD cells, but undetectable in float-up fractions from undifferentiated CADs (Fig 7B) despite the fact that levels of KIF5C protein expression were only moderately diminished in undifferentiated CAD cells compared to differentiated cells (Fig 5C). In contrast mCitrine-KIF1A was detectable on PRV capsid associated particles at similar frequency whether they had been prepared from differentiated or undifferentiated CAD cells (Fig 7C).

### Loss of gE/gI and US9p does not affect the efficiency of dynein recruitment

**Fig 8:**
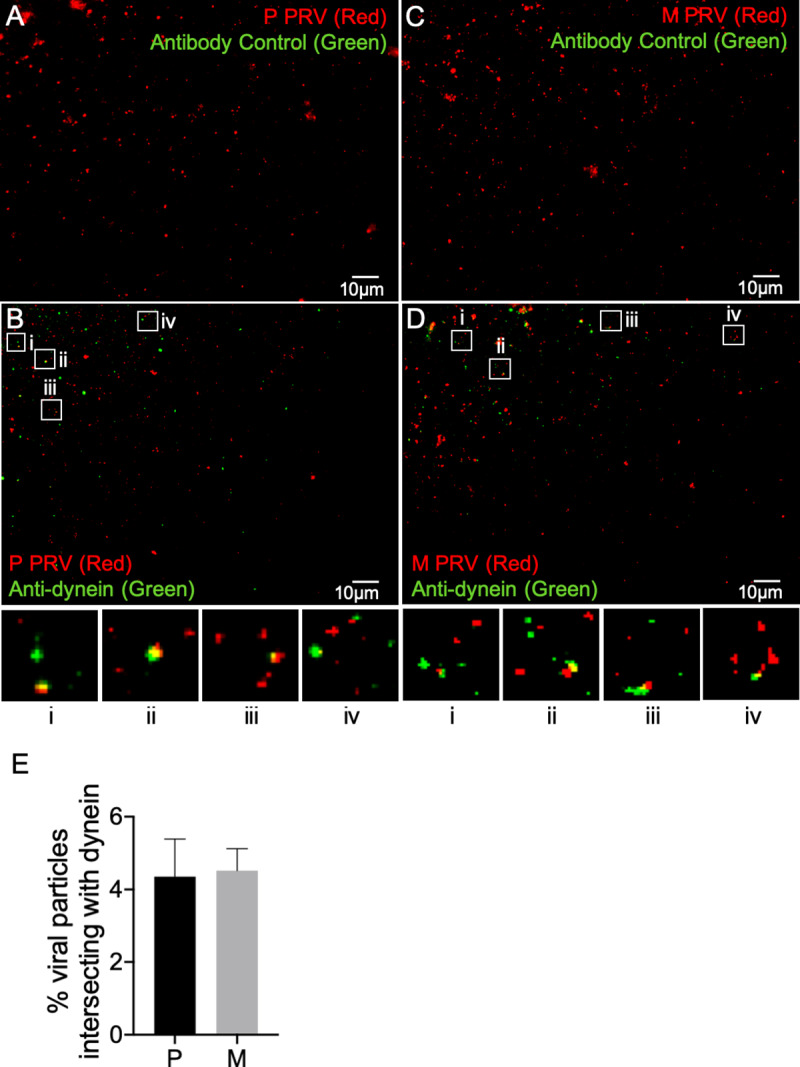
Loss of gE/gI-US9p does not affect the efficiency of dynein recruitment. Float-up fractions from P or M PRV-infected differentiated CAD cells were immunostained with a mouse monoclonal anti-dynein antibody or the antibody omitted as control (indicated by text in each panel), imaged in the red (mCherry-tagged capsids) and green (anti-mouse antibody immunofluorescence) channels and images merged. **(A, B)** Fields of float-up particles from a P PRV infection stained in the absence or presence of anti-dynein IgG, respectively. **(C, D)** Fields of float-up particles from a M PRV infection stained in the absence or presence of anti-dynein IgG, respectively. Four boxed regions (i-iv) in panels B and D were magnified 3-fold and are shown at the bottom of each panel. The individual channel images used to construct the merged panels in A-D are shown in inverted black and white in S6 Fig and S7 Fig. **(E)** Numbers of P and M red fluorescent particles that exhibit anti-dynein staining as a percent of total particles in the field, derived from images similar to those in (B) and (D). Plotted values are mean and standard deviation from the mean for 7,914 P particles (imaged from 14 independent microscopic fields) and 7,932 M particles (imaged from 13 independent microscopic fields).

Increased levels of minus end-directed motion, and greater frequency of reversal in direction of trafficking (Fig 3) are consistent with our finding that M PRV particles recruit diminished levels of kinesins KIF1A and KIF5C (Fig 4 and Fig 6). However an alternative, and not mutually exclusive, possibility is that loss of gE/gI-US9p enhances the binding of dynein to egressing PRV particles. To test this we prepared float-up fractions from M or P PRV-infected differentiated CAD cells and immunostained them in the absence (Fig 8A, 8C) or presence (Fig 8B and 8D) of an anti-dynein antibody. As quantitated in Fig 8E we observed no significant difference in the frequency with which dynein decorated P or M viral particles. We conclude that loss of gE/gI-US9p does not affect the numbers of PRV particles able to recruit dynein motors. However we cannot exclude the possibility that the *activity* of PRV-bound dynein is derepressed by the loss of gE/gI-US9p either directly, or indirectly due to the absence of gE/gI-US9p-bound/activated kinesins [49, 50].

## Discussion

The alphaherpesvirus-encoded membrane proteins gE/gI and US9p are important for the anterograde spread of PRV and HSV-1 and are required for the efficient delivery of viral particles from the cell body into, or along, axons and neurites [1, 3, 10]. Several models exist to explain how they accomplish this task, including roles in generation of the enveloped cytoplasmic virions for subsequent trafficking to the axon [15, 36], provision of motor-loading platforms on the surfaces of cytoplasmic organelles [15, 24] and direct binding of the kinesin motor KIF1A to trafficking virions *via* US9p [27, 28]. The identity of the kinesin motors used to traffic alphaherpesvirus particles into and along axons is also the topic of some debate [3, 26, 30]. Although PRV US9p interacts with KIF1A, and PRV virions are associated with KIF1A during anterograde transport down the axon [27], loss of US9p only diminishes the numbers of PRV particles able to reach the axon, not their trafficking within it [29] and HSV-1 particles appear to utilize members of the KIF5 family for their axonal trafficking [30].

To attempt to clarify some of these issues we have quantitatively examined the assembly, trafficking properties and motor association of a PRV strain (P) and a derivative (M) deleted for the genes encoding gE/gI and US9p. Following PRV replication in PK15 porcine epithelial cells, or differentiated mouse neuronal CAD cells, total cellular cytoplasmic extracts (PNS) and buoyant float-up organellar-enriched fractions were found to contain similar numbers of P and M capsid-associated viral particles (Figs 1 and 2). Because our float-up conditions select for membrane-associated capsids whether they are sealed into mature envelopes or simply docked to the surface of organelles [45, 51, 52] it remained possible that Δ(gE/gI-US9p) capsids were able to attach to organelles but nevertheless exhibit an envelopment defect. We therefore tested PFU yields in each of these fractions and found that P and M strains generated similar numbers of infectious particles, inconsistent with an envelopment or other maturation deficiency (Figs 1 and 2). We conclude that loss of gE/gI-US9p has no significant effect upon the numbers of enveloped organelle-associated PRV virions assembled in these epithelial or neuronal cell lines under our conditions. In this respect PRV appears to differ from HSV-1, since simultaneous loss of gE and US9p resulted in the accumulation of partially enveloped and non-enveloped HSV-1 capsids in the cell body of infected CAD cells (6 fold and 12 fold increases, respectively), and a 5 to 7 fold decrease in viral titer [15]. Similar results were obtained for HSV-1 replicating in human SK-N-SH neurons [15].

Since assembly of organelle-associated enveloped PRV virions was unimpaired we next tested the characteristics of microtubule-mediated motility of P and M particles using our *in vitro* motility assay [44–46]. An advantage of this *in vitro* approach is that it permits measurement of the properties of PRV particles lacking US9p, virions whose motility in living neurons is difficult to analyze because they are largely incapable of leaving the cell body and entering the axon [26]. We found that approximately 85% of motile P PRV-containing transport organelles prepared from differentiated CAD neurons moved to the plus ends of microtubules (Fig 3A). However, in the absence of gE/gI-US9p the numbers of plus end-directed motions were reduced while minus end directed transport was increased; overall Δ(gE/gI-US9p) viral particles came to exhibit an approximately equal probability of traffic in either direction (Fig 3A). The trafficking properties of individual particles were also affected since Δ(gE/gI-US9p) virions showed an increased likelihood of switching direction during transport (Fig 3B). We obtained similar results for viral particles prepared from epithelial PK15 cells, suggesting that gE/gI and US9p function to recruit kinesins even in non-neuronal cells, and that that this recruitment extends beyond neuron-specific kinesins such as KIF5C (Fig 3C and 3D). A role for gE/gI or the gE/gI-US9p complex in trafficking of PRV particles through the epithelial cell cytoplasm is consistent with the fact that gE/gI are required for efficient sorting of HSV-1 to the basolateral surfaces of epithelial cells [38, 53].

Alphaherpesviruses have long been known to exhibit bidirectional transport during overall anterograde spread [3, 47], suggesting that both plus and minus end-directed motors are present on the particle. The presence of multiple molecular motors on microtubule-bound cargo greatly enhances the processivity of transport, even when those motors act in opposed directions [50]. Moreover, it is increasingly clear that bidirectional motion is a common feature of microtubule-directed organellar transport, and that the overall net direction of trafficking results from a complex process of balancing and cross-talk between opposed motors [49, 54]. Our data suggest that loss of gE/gI-US9p shifts the “tug of war” between opposed PRV-associated molecular motors, altering the balance from plus-end motion in favor of minus-end and bidirectional traffic (Fig 3).

Enhanced minus end-directed and bidirectional traffic could result from greater binding or activation of dynein and/or decreased recruitment and activation of kinesins. We found that loss of gE/gI-US9p had no measurable effect upon the efficiency of colocalization of PRV particles with dynein (Fig 8), but greatly reduced the numbers of virions associated with kinesins KIF1A and KIF5C (Fig 4, Fig 6). The simplest explanation for our motility data is therefore that gE/gI-US9p normally serves to recruit or activate kinesin(s) to drive efficient plus end-directed PRV traffic and to suppress minus end and bidirectional motion (Fig 3), despite the presence of opposed minus-end motors on the particle. The gE/gI-US9p complex could achieve this by elevating the copy number of kinesin molecules on the organelle containing the virion, by stimulating the specific activity of the kinesin motor or by suppressing minus end motor function [49, 54]. Evidence for the latter two possibilities has recently been presented by Scherer and colleagues [26] who demonstrated that loss of gI from the gE/gI-US9p complex reduces the velocity of anterograde trafficking PRV particles and results in increased retrograde traffic of axonal virions. That study concluded gI (directly, or in cooperation with other components of the gE/gI-US9p complex) can help accelerate the velocity of the KIF1A motor and/or might inhibit the activity of dynein associated with the viral particle [26].

The finding that M PRV particles prepared from differentiated CAD neurons fail to recruit KIF1A (Fig 4) is consistent with the hypothesis that gE/gI-US9p provides a KIF1A-receptor activity [26–28]. However, this study is the first to demonstrate that deletion of gE/gI-US9p also abolishes viral association with KIF5C (Fig 6) despite the abundant expression of this motor in P and M infected CAD cells (Fig 5). This result was unexpected since the PRV gE/gI-US9p complex does not appear to interact with KIF5 motors [26], although the US9p protein of HSV-1 has been reported to associate with the C-terminal tail of KIF5B [35].

We believe the simplest explanation for our data is that gE/gI-US9p-dependent KIF1A recruitment is a *prerequisite* for subsequent association of PRV particles with KIF5C. In this model KIF1A (which can associate with P PRV cargo even in undifferentiated CAD cells, Fig 7C) mediates the trafficking of PRV particles to an organelle or intracellular location at which KIF5C is subsequently recruited. Our data suggest that KIF5C *replaces* KIF1A at this location, since we rarely observe both motors on the same particle (Fig 7A). Since KIF5C does not associate with PRV particles in undifferentiated CAD cells (despite abundant expression of KIF5C, Fig 5C) the location or biological environment in which the motors are exchanged must be dependent upon neuronal differentiation (Fig 7B). The replacement of KIF1A by KIF5C could be facilitated *via* gE/gI-US9p-stimulated degradation of KIF1A by the proteosome, as recently described in PC12 cells and rat SCG neurons [55]. Although we observed equivalent levels of KIF1A protein in float-up fractions prepared from uninfected, P-infected and M-infected differentiated CAD cells (Fig 4A) we have not examined total levels of KIF1A in whole cell extracts and it remains possible that KIF1A in these cells is similarly subject to proteosomal degradation. KIF1A turnover appears to occur in the axon [55] and we speculate that KIF1A/KIF5C motor exchange occurs at the axon initial segment (AIS) [3, 56, 57] since kinesin-1, but not kinesin-3, motors prefer to bind the highly modified, stabilized MTs found at this location [57–59]. KIF5C recruitment in the AIS would be consistent with our finding that CAD cells must be differentiated in order to load KIF5C onto the PRV particle. Alternatively, KIF5C recruitment could occur in the cell body but then would presumably require post translational modifications, or expression of additional factors, that are specific to the differentiated CAD cell.

Alternative explanations exist for our finding that PRV associates with KIF1A or KIF5C motors, but not both simultaneously. One possibility is that KIF5C mediates transport from the cell body to the axon, and KIF1A subsequently replaces it to drive traffic of axonal PRV particles. Alternatively, KIF5C or KIF1A recruitment might represent parallel, redundant, pathways for PRV transport into and along the axon. However a problem with both of these models is that they fail to explain why Δ(gE/gI-US9p) PRV particles are unable to recruit the KIF5C motor.

## Materials and methods

### Cells and viruses

PK15 cells were maintained in Roswell Park Memorial Institute (RPMI) 1640 medium supplemented with 10% fetal calf serum (FCS, Peak Serum Inc.) and 1% penicillin-streptomycin (Gibco Laboratories). CAD cells were cultured in Dulbecco’s Modified Eagle Medium (DMEM)/F12 supplemented with 15mM HEPES (Gibco Laboratories) supplemented with 10% FCS and 1% penicillin-streptomycin. For CAD cell differentiation cells were grown to 70% confluence, washed twice with phosphate-buffered saline (PBS; 137 mM NaCl, 2.7 mM KCl, 10 mM Na_2_HPO_4_, 1.8 mM KH_2_PO_4_, pH 7.4) to deplete serum incubated in differentiation medium comprised of DMEM/F12 supplemented with 10μg/ml Transferrin (Sigma-Aldrich), 50ng/ml Sodium Selenite (Sigma-Aldrich) and 1% penicillin-streptomycin. Differentiation medium was replaced after 3 days of differentiation and cells were infected after 5 days of differentiation. For expression of the mCitrine-KIF1A fusion protein undifferentiated CAD cells were transfected with the appropriate expression construct using Lipofectamine 3000 (Life Technologies) according to the manufacturer’s protocol. After three days the transfected CAD cells were then either infected with PRV or were transferred to differentiation medium for 5 days then infected as detailed above.

PRV-GS4284 and PRV-GS6090 were derived from the pBecker3 infectious clone [60] using a two-step red recombination protocol [61]. PRV-GS4282 encodes the mCherry fluorescent protein as a translational fusion to the pUL25 capsid protein and was previously described [62]. PRV-GS6090 encodes the same pUL25/mCherry fusion and a deletion spanning the US7, US8, and US9 genes. The deletion was made with the primer pair: 5’ GGTCCCGCGGACACCGACGAGCTAAAAGCGCAGCCCGGTCCGTAG//TCGCTGTCGGCGCTGAGGATGACGACGATAAGTAGGG and 5’ CTACACGTGCCGGGCGATGATGCCCCCGATCAGCGCCGACAGCGA//CTACGGACCGGGCTGCAACCAATTAACCAATTCTGATTAG (in each primer the deletion site is indicated with a //, and the 3’ sequence complementary to the pEP-KanS2 template is underlined). The deletion begins immediately following the UL6 stop codon and ends in the 3’ coding region of US9 such that last 14 codons of US9 remain intact but without an in-frame start codon. This design places the US9 polyadenylation signal in proximity to US6. The pGS4282 and pGS6090 infectious clones were transfected into PK15 epithelial cells to produce PRV-GS4284 and PRV-GS6090, as previously described [63]. Working stocks were made by an additional passage through PK15 cells and titers were determined on PK15 cells as previously described [64] or on Vero cells grown in DMEM supplemented with 10% Newborn Calf Serum (NCS) and 1% penicillin-streptomycin. Both viruses are kind gifts from Dr. Gregory A. Smith.

### Preparation of post nuclear supernatants and gradient float-up fractions

PK15 cells were infected with PRV strains GS4284 or GS6090 at a multiplicity of infection (MOI) of 10 for 1 hr at 37ºC. They were then acid-washed to inactivate unpenetrated virus as previously described [65], overlaid with fresh prewarmed media and incubated at 37ºC for 16 hr. Infected cells were then washed once with ice-cold MEPS buffer (5 mM MgSO_4_, 5 mM EGTA, 0.25 M sucrose, 35 mM piperazine-N,N′-bis [2-ethanesulfonic acid] [PIPES] pH 7.1), scraped up and resuspended in MEPS buffer containing 2 mM phenylmethylsulfonyl fluoride (PMSF), 2% (vol/vol) protease inhibitor cocktail (Sigma Aldrich, Cat# P8465) and 4 mM dithiothreitol (DTT). A postnuclear supernatant (PNS) was then prepared as previously described [45, 46], adjusted to a final sucrose concentration of 1.4 M, overlaid with layers of 1.2 M and 0.25 M sucrose in MEPS buffer then subjected to density gradient centrifugation to isolate a membrane-associated buoyant float-up virus fraction as previously described [45, 46]. Undifferentiated or differentiated CAD cells were infected for 1 hr at 37ºC, covered with fresh prewarmed media and incubated for 20 hr. Infected cells were collected, washed twice with ice-cold MEPS buffer and PNS and float-up extracts prepared as described above. In all cases PNS and float up fractions were flash frozen in liquid nitrogen in small aliquots prior to storage at −80ºC.

### Fluorescence imaging, immunostaining and data analysis

Samples of PNS and float-up extracts were spotted onto glass coverslips precoated with 10 μg/ml Poly-L-Lysine (Sigma-Aldrich) and allowed to attach for 2 hr. They were then fixed with 4% paraformaldehyde for 30 min at room temperature (RT) and washed with PBS. For direct fluorescence imaging coverslips were then immediately mounted using ProLong Diamond Antifade agent (Life Technologies, Cat# P36961). For immunostaining, coverslips were incubated in blocking buffer (2 mg/ml Bovine serum albumin [BSA] in PBS) for 30 mins. For detection of KIF5C coverslips were incubated for 1 hr at RT with rabbit IgG anti-KIF5C (abcam, Cat# ab193352) or an equal mass of control rabbit IgG (Sigma-Aldrich, Cat# I5006-10mg), washed with blocking buffer then incubated with Alexa Fluor^TM^ 405-labeled goat anti-rabbit IgG (Life Technologies, Cat# A31556) for 1h at RT, then washed and mounted for imaging as described above. For detection of dynein coverslips were prepared and incubated in an identical manner to the above except that primary and secondary antibodies were mouse anti-dynein monoclonal IgG clone 74.1 (MilliporeSigma, Cat# MAB1618) and Alexa Fluor^TM^ 405-labeled goat anti-mouse IgG (Life Technologies, Cat# A31553).

Imaging (other than of motile viral particles, see below) was performed in the Analytical Imaging Facility (AIF) of the Albert Einstein College of Medicine. Images were collected on a Zeiss Axio Observer CLEM inverted microscope with a 63 X oil-immersion, 1.4-numerical aperture objective. All Images were saved in Zeiss AxioVision ZVI (Zeiss Vision Image) format using AxioVision Rel. 4.8.2 Image Acquisition and Management software. NIH-ImageJ software was used for image analysis.

Virus particle numbers in fluorescent fields were counted using the open source software Fiji (http://fiji.sc) [66, 67]. The images of microscopic fields were dragged to the Fiji tool bar and converted to Binary. The same image threshold was applied to all images derived from a single experiment. The number of virus particles in each field was calculated using the Fiji plugin “Analyze Particles”.

### Analysis of microtubule-mediated motility of viral particles

Viral particles were prepared on float-up density gradients as described above. Preparation of fluorescent microtubules and analysis of viral particle motility in microscopic imaging chambers was by modification of methods previously described [44–46]. Lyophilized, non-fluorescent porcine brain tubulin (Cytoskeleton Inc., Cat# T240-A) was adjusted to 10 μg/μl in Buffer 1 (1 mM EGTA, 1 mM MgCl_2_, 1mM DTT, 1 mM GTP, 80 mM K_2_ PIPES pH 7.4), clarified by centrifugation at 20,000 x g for 10 min then small aliquots flash frozen in liquid nitrogen. For microtubule preparation these aliquots were thawed, placed on ice, and 80 μg of unlabeled tubulin mixed with 20 μg HiLyte 647-labeled porcine brain tubulin (Cytoskeleton Inc., Cat# TL670M-A) to yield a 1:4 molar ratio of labeled: unlabeled tubulin at a concentration of 12.5 μg /μl. Half of this mixture was incubated at 37ºC for 5 min to generate polymerization "seeds" which were sheared by repeated pipetting, then mixed with the remaining unpolymerized 1:4 tubulin mixture. This "growth" mixture was allowed to polymerize for 6 min at 37ºC and polymerization terminated by addition of Buffer 1 supplemented with 20 μM Taxol. The microtubules were collected by pelleting at 57,000 x g for 1 min in a Beckman Airfuge (Model # 350624, rotor A-100/30) at RT then resuspended in Buffer 1 supplemented with 20 μM Taxol and stored in darkness at RT for up to 1 week.

For motility assays 4 μl volume optical chambers were constructed as described [68] with coverslips coated in 10 μg/ml diethylaminoethyl (DEAE)-dextran followed by extensive washing in H_2_O. Polymerized microtubules (prepared as described above) were diluted 1:25 in Buffer 1 supplemented with 20 μM Taxol and added to each optical chamber. After 3 min chambers were washed once in assay buffer-casein (AB-C) comprised of motility assay buffer (AB; 85 mM K gluconate, 5 mM Na gluconate, 5 mM MgCl_2_, 1 mM EGTA, 2 mg/mL BSA, 4 mM DTT, 10 μM Ouabain, 20 μM Taxol, 11 mM Hepes, 9 mM Tris pH 7.4) supplemented with 5 mg/ ml solubilized casein, and placed on ice. To bind viral particles to the microtubules viral float-up fractions (prepared as described above) were thawed, diluted as required in AB-C, added to the chambers and incubated for 15 min on ice. The chambers were then washed once in AB and stored on ice until needed. Motility experiments were initiated by allowing chambers to warm to RT for 1 min, transferring them to a 35ºC prewarmed microscope stage and initiating multi-channel fluorescence image capture at 1 frame per 5.5–6.5 sec for 20 frames (S1 Fig, S2 Fig), concurrent with addition of 100 μM ATP. Control and experimental samples were alternated during the image capture process.

Polarity-marked microtubules for determination of plus or minus end-directed motion were prepared by modification of methods previously described [54, 69]. Non-fluorescently-labeled porcine brain tubulin (8.53 μg/μl) and HiLyte 647-labeled porcine brain tubulin (1.07 μg/μl) were allowed to polymerize at 37ºC for 3.5 min to generate dimly fluorescent “seed microtubules” and polymerization terminated by addition of prewarmed Buffer 1 supplemented with 10 μM Taxol. The mixture was vortexed for 10 min to shear the seeds and seeds pelleted by centrifugation at 57,000 x g for 3 min in a Beckman Airfuge (see above) at RT. The pellet was washed once with prewarmed Buffer 1, resuspended in prewarmed Buffer 1 and 29 μg (at 9.6 μg/μl) sheared, dimly fluorescent microtubule seeds added to a mixture of 17.3 μg (at 1.73 μg/μl) unlabeled and 34.7 μg (at 3.47 μg/μl) HiLyte 647-labeled tubulin. This mixture was then immediately incubated for 15 min at 37ºC to polymerize brightly fluorescent microtubules from the dim seeds. Polymerization was terminated by addition of prewarmed Buffer 1 supplemented with 10 μM Taxol. Polarity marked microtubules were added to optical chambers without additional pelleting. Imaging studies of motile virions were as described above except that assays were always performed within one hour of microtubule preparation.

Virion motility was imaged on an Olympus iX71 microscope and 60x 1.4 NA oil lens using Metamorph Software (Molecular Devices LLC, Sunnyvale, CA) equipped with a Sedat multiband filter dichroic, automated X-Y-Z stage (Applied Scientific Instrumentation), CoolSnap HQ cooled CCD camera (Photometrics), DG-4 xenon excitation lamp (Sutter Instrument Co., Navoto, CA) with automated filter switching appropriate for Dapi, FITC, Rhodamine, and Cy5 fluorescence and bright field channels. *In vitro* motility was scored by automated analysis using macros written for Fiji / ImageJ (http://fiji.sc) [66, 67] that included a spot enhancing filter [70]. The macro involved imaging processing and segmenting the microtubules, triangle autothresholding to create a binary microtubule mask image, autothresholding and automated counting of virus particle spots within the mask, scoring movement as loss of viral particle fluorescence intensity at the spot location, and visual confirmation of accuracy. Directional and bi-directional movement was scored manually as processive movements greater than 4 pixels (0.84 microns).

### Western blotting

Whole cell extracts, PNS samples or gradient float-up fractions were subjected to SDS PAGE electrophoresis on 7.5% gels then transferred to a polyvinylidene difluoride membrane as previously described [71], blocked using a solution of 5% dried milk in Tris-buffered saline tween (TBST; 137 mM NaCl, 0.1% w/v Tween-20, 20 mM Tris.Cl pH 7.4) then incubated with the appropriate primary antibody using dilutions as per suppliers recommendation. Anti-KIF1A antibody (Cat# ab180153) and anti-KIF5C antibody (see immunostaining section) were obtained from abcam and anti-β-Actin antibody (Cat# sc47778) from Santa Cruz Biotechnology, Inc. After washing, membranes were incubated with peroxidase-conjugated anti-rabbit or anti-mouse secondary antibodies (MilliporeSigma) and bound antibody detected with an enhanced chemiluminescence (ECL) substrate (PerkinElmer). KIF5C Western blot band intensity was quantitated using Fiji / ImageJ (http://fiji.sc) [66, 67] and plotted as the ratio of KIF5C band density to that of the loading control β-Actin.

## Supporting information

S1 Fig Movie of *in vitro* P PRV particle motion along polarity-marked microtubules. Representative example of particle motions used to generate the data in Fig 3. P PRV (mCherry red fluorescent particles) move along green fluorescent microtubules. The dimly fluorescent “seed” regions used to grow the microtubules are indicated by white arrowheads, and identify the minus ends of the microtubules. Images were taken at 6.5 sec intervals. Scale bar: 5 μm.
(AVI)

S2 Fig Kymograph of *in vitro* PRV particle motion along polarity-marked microtubules. Individual PRV particle motions from *in vitro* movies were visualized by drawing a line along the microtubules from their minus to plus end and projecting viral particle mCherry fluorescence intensity through time using the Fiji/ImageJ reslice function. The X-axis represents 2 dimensional distance, the Y-axis represents time, with scale bars shown. Polarity-marked microtubules are represented at top of figure, with dimly fluorescent seeds indicated by double dashed lines (—) and the plus (+) and minus (-) ends of the microtubule indicated. The motion of 28 randomly selected P (left) and 27 randomly selected M (right) PRV particles are shown over a 130 sec time period in 6.5 sec intervals. The starting positions of the particles (location at time = 0) have been aligned.
(TIF)

S3 Fig Raw image data used in Fig 4B and 4C. The individual red and green images used to generate the merged panels in Fig 4B and 4C are shown in inverted black and white. Panel labeling corresponds to the panels in Fig 4.
(TIF)

S4 Fig Raw image data used in Fig 6A and 6C. The individual red and green images used to generate the merged panels in Fig 6A and 6C are shown in inverted black and white. Panel labeling corresponds to the panels in Fig 6.
(TIF)

S5 Fig Raw image data used in Fig 6B and 6D. The individual red and green images used to generate the merged panels in Fig 6B and 6D are shown in inverted black and white. Panel labeling corresponds to the panels in Fig 6.
(TIF)

S6 Fig Raw image data used in Fig 8A and 8C. The individual red and green images used to generate the merged panels in Fig 8A and 8C are shown in inverted black and white. Panel labeling corresponds to the panels in Fig 8.
(TIF)

S7 Fig Raw image data used in Fig 8B and 8D. The individual red and green images used to generate the merged panels in Fig 8B and 8D are shown in inverted black and white. Panel labeling corresponds to the panels in in Fig 8.
(TIF)
