# Supplementary figures and images for: Deletion of the Pseudorabies Virus gE/gI-US9p complex disrupts kinesin KIF1A and KIF5C recruitment during egress, and alters the properties of microtubule-dependent transport in vitro
Source: PLoS Pathog. 2020 Jun 8;16(6):e1008597. doi: 10.1371/journal.ppat.1008597 (PMC7302734; doi:10.1371/journal.ppat.1008597)

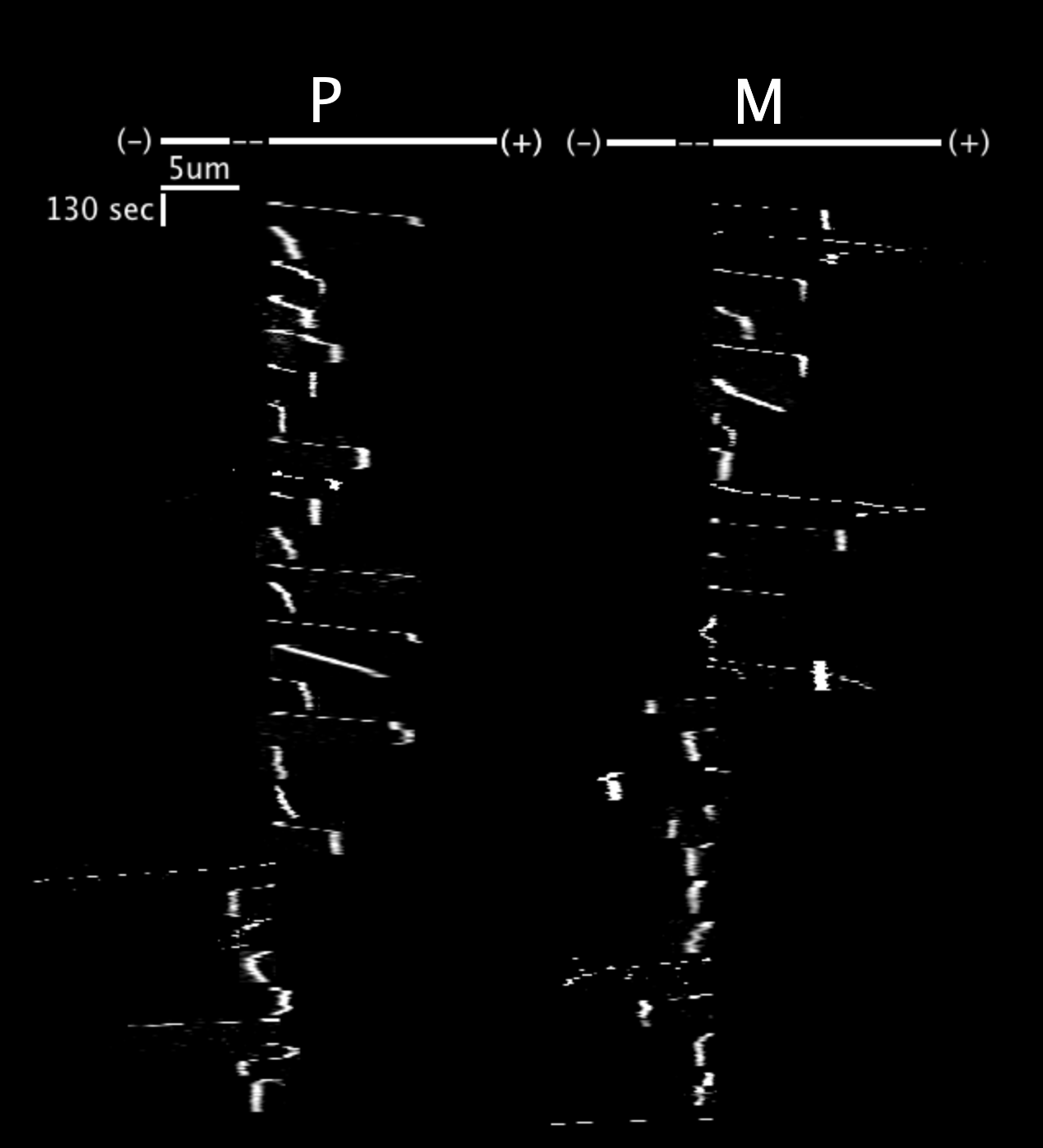

Supplement: S2 Fig — Individual PRV particle motions from in vitro movies were visualized by drawing a line along the microtubules from their minus to plus end and projecting viral particle mCherry fluorescence intensity through time using the Fiji/ImageJ reslice function. The X-axis represents 2 dimensional distance, the Y-axis represents time, with scale bars shown. Polarity-marked microtubules are represented at top of figure, with dimly fluorescent seeds indicated by double dashed lines (—) and the plus (+) and minus (-) ends of the microtubule indicated. The motion of 28 randomly selected P (left) and 27 randomly selected M (right) PRV particles are shown over a 130 sec time period in 6.5 sec intervals. The starting positions of the particles (location at time = 0) have been aligned. (TIF) [file ppat.1008597.s002.tif]

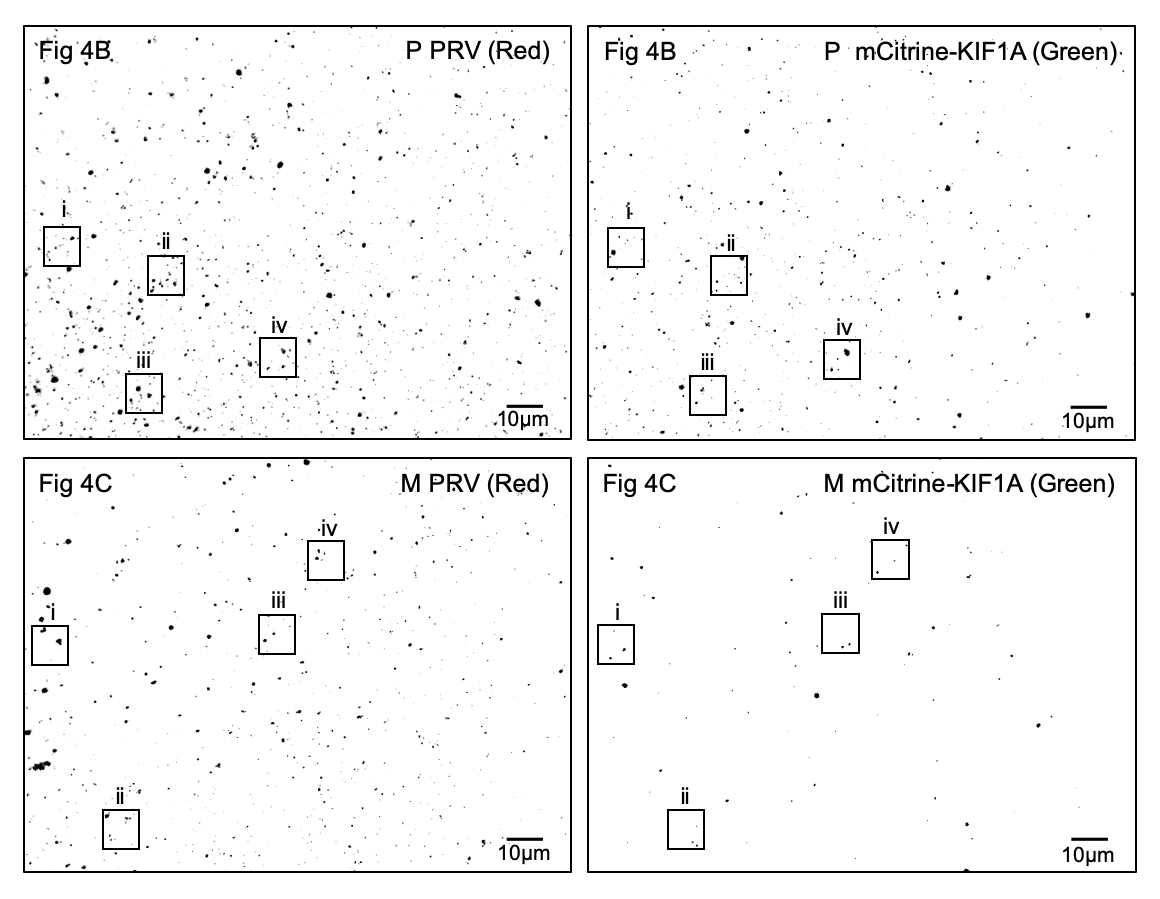

Supplement: S3 Fig — The individual red and green images used to generate the merged panels in Fig 4B and 4C are shown in inverted black and white. Panel labeling corresponds to the panels in Fig 4. (TIF) [file ppat.1008597.s003.tif]

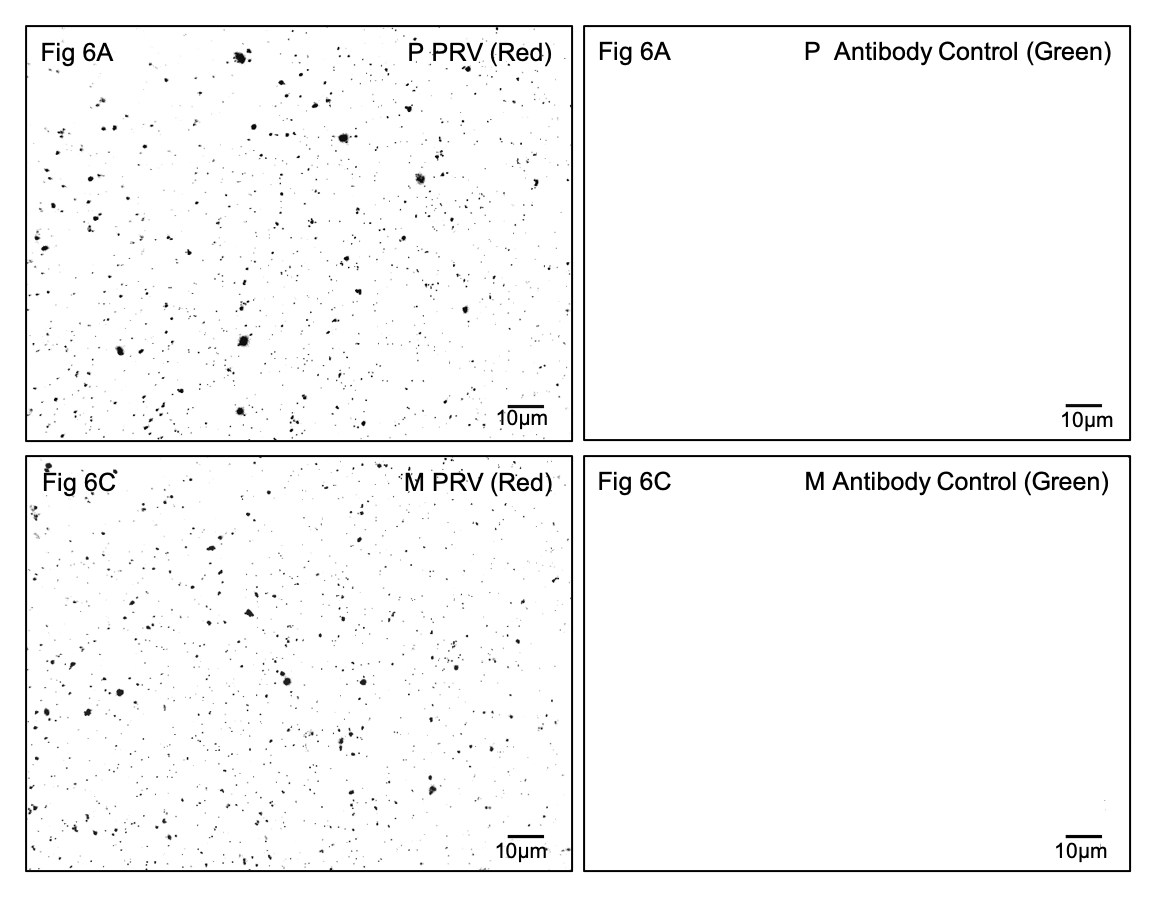

Supplement: S4 Fig — The individual red and green images used to generate the merged panels in Fig 6A and 6C are shown in inverted black and white. Panel labeling corresponds to the panels in Fig 6. (TIF) [file ppat.1008597.s004.tif]

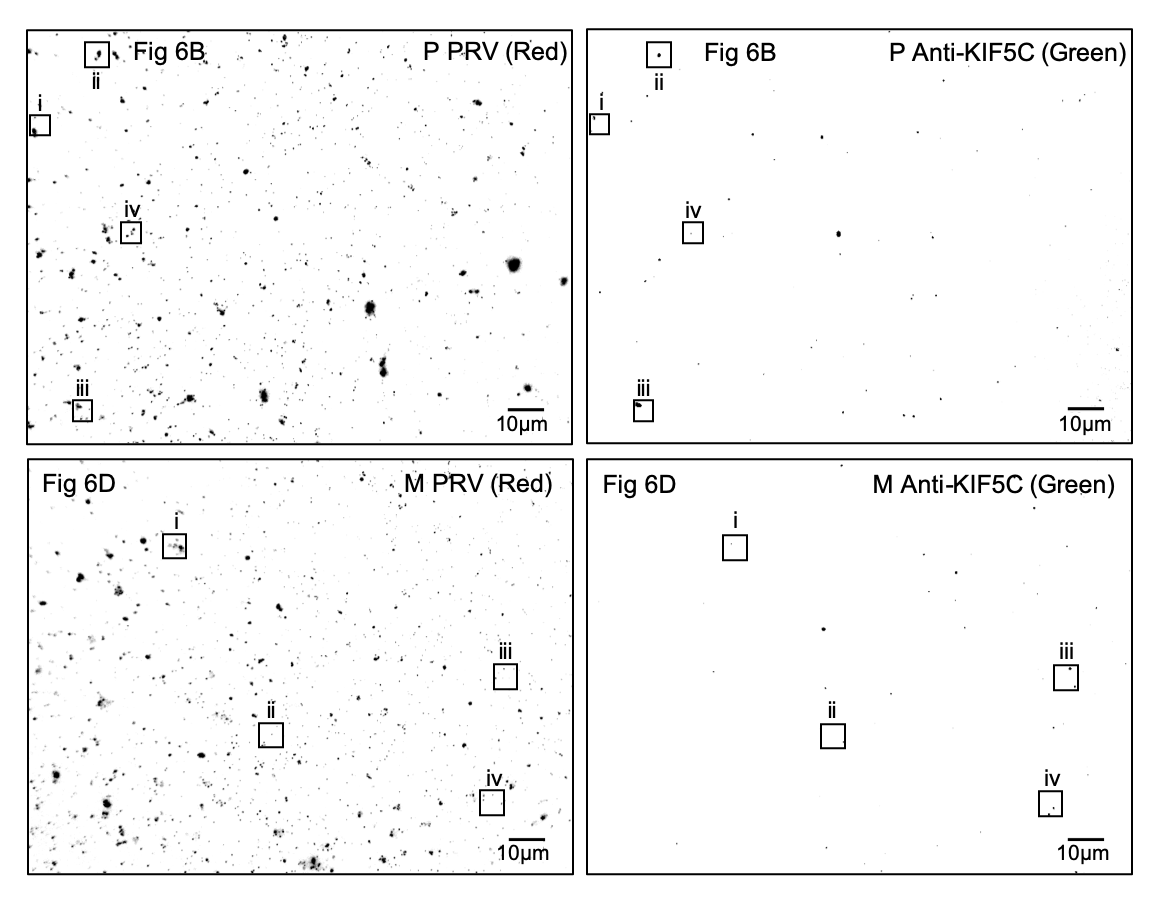

Supplement: S5 Fig — The individual red and green images used to generate the merged panels in Fig 6B and 6D are shown in inverted black and white. Panel labeling corresponds to the panels in Fig 6. (TIF) [file ppat.1008597.s005.tif]

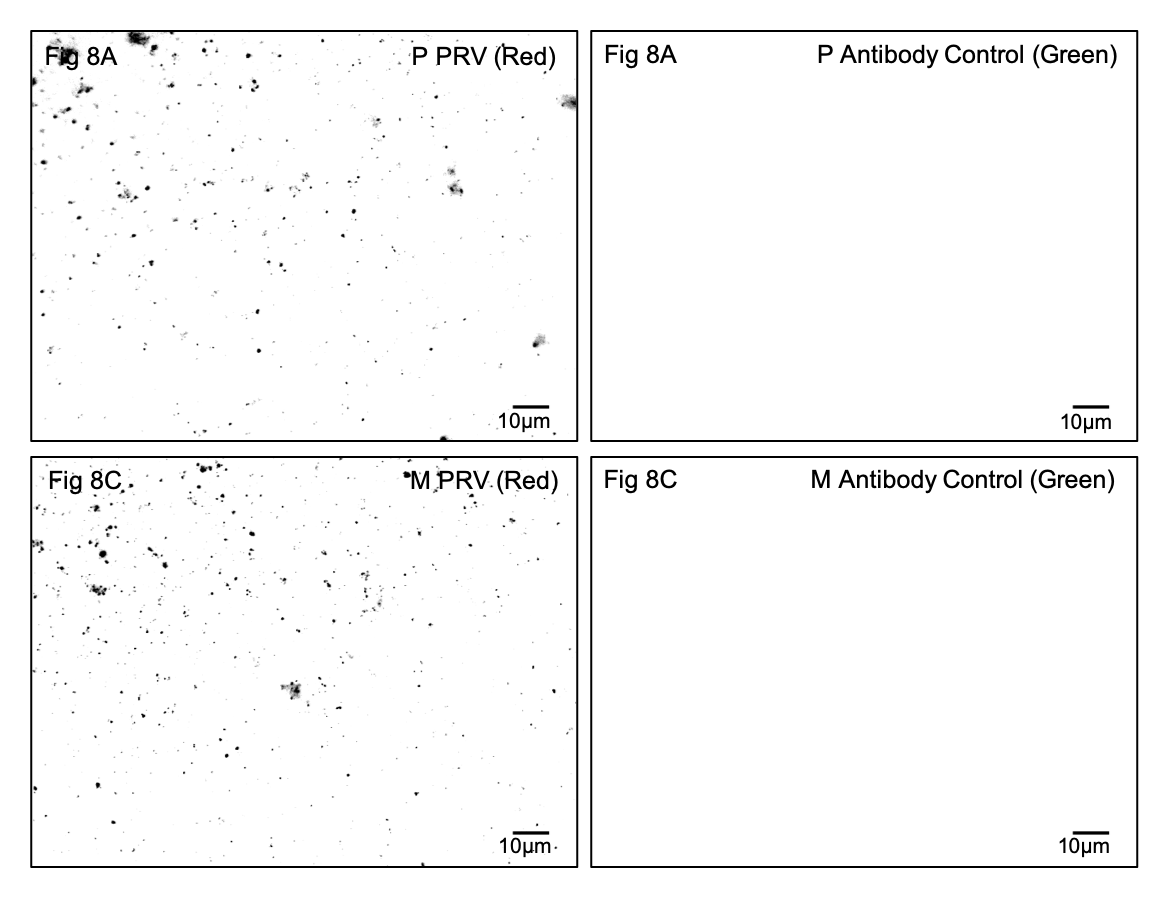

Supplement: S6 Fig — The individual red and green images used to generate the merged panels in Fig 8A and 8C are shown in inverted black and white. Panel labeling corresponds to the panels in Fig 8. (TIF) [file ppat.1008597.s006.tif]

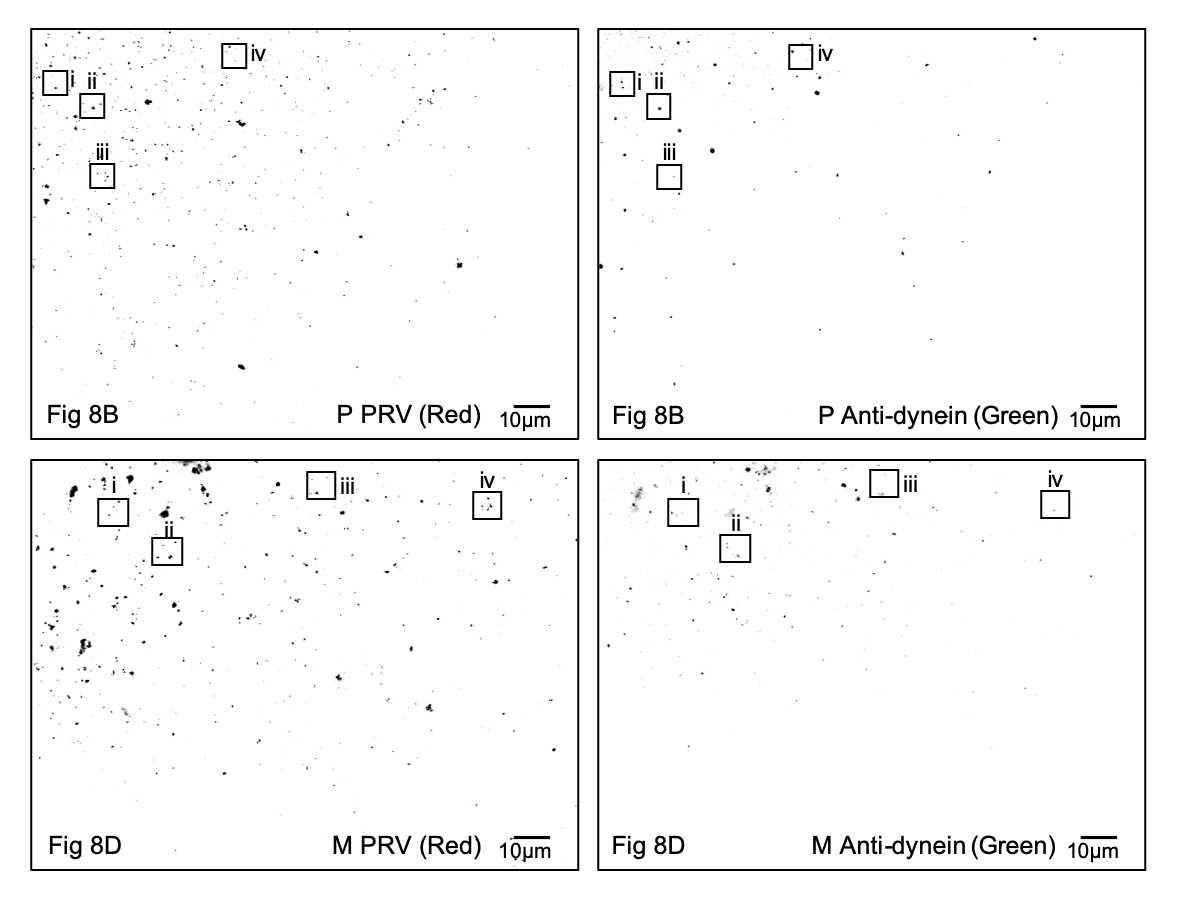

Supplement: S7 Fig — The individual red and green images used to generate the merged panels in Fig 8B and 8D are shown in inverted black and white. Panel labeling corresponds to the panels in in Fig 8. (TIF) [file ppat.1008597.s007.tif]
